# CISCS: Classification of inter-class similarity based medicinal plant species groups with machine learning

**DOI:** 10.1016/j.mex.2025.103652

**Published:** 2025-09-30

**Authors:** N. Shobha Rani, Bhavya K R, I. Jeena Jacob, Pushpa B. R, Bipin Nair BJ, Akshatha Prabhu

**Affiliations:** a MURTI Research Centre, Smart Agriculture Lab, Department of Artificial Intelligence and Data Science, GITAM School of Technology, Bengaluru, GITAM (Deemed to be) University, India; b MURTI Research Centre, Smart Agriculture Lab, Department of Computer Science and Engineering, GITAM School of Technology, Bengaluru, GITAM (Deemed to be) University, India; c Department of Computer Science, School of Computing, Amrita Vishwa Vidyapeetham, Mysuru, India

**Keywords:** Medicinal plant classification, Deep learning, Intra-class variation, High inter-class similarity, Indian plant species

## Abstract

The reliable classification of medicinal plant species plays a vital role in ensuring their quality, authenticity, and safe use in healthcare. However, existing methods often face difficulties when species exhibit strong visual similarities or when datasets are imbalanced, which limits their effectiveness in practice. Although deep learning models such as ResNet18 and VGG16 have proven influential in image recognition tasks, our experiments showed that they tended to overfit, with validation losses reaching 42.99 % and test accuracy falling to 73.99 % in certain groups. To overcome these challenges, we introduce a multi-level fusion feature model that combines 3D normalized color histograms, extended uniform Local Binary Patterns (LBP with *P* = 24, *R* = 3), multi-orientation Gabor filters, and Histogram of Oriented Gradients (HOG). This approach captures a richer set of visual cues by bringing together global color statistics, detailed textures, frequency-domain patterns, and shape descriptors. We incorporate SMOTE-based synthetic augmentation to address further class imbalance, which helps balance feature distributions across categories. We employ a soft-voting ensemble of machine learning classifiers for classification and use cosine similarity metrics to capture inter-class relationships better. Tests on Indian medicinal plant datasets show that our model consistently outperforms deep learning baselines, reaching 100 % accuracy in Group 1, 95.82 % in Group 3, and over 90 % in other groups. These results suggest that the proposed model offers a more robust and computationally efficient solution for plant species classification, particularly under conditions of high inter-class similarity and dataset imbalance.

• The proposed domain-specific model can be applied explicitly to Indian plant species groups exhibiting high inter-class visual similarities through a novel feature fusion strategy.
• The proposed multi-level feature fusion method's innovation integrates 3D normalized color histograms, extended uniform LBP (*P* = 24, *R* = 3), multi-orientation Gabor filters, and HOG features to capture the color, texture, and shape characteristics.
• The proposed work offers a scalable ensemble framework for inter-class similarity analysis by combining SMOTE-based class balancing, feature normalization, and a soft-voting ensemble of diverse classifiers that support biodiversity and ecological studies.

## Graphical abstract

**Figure:**
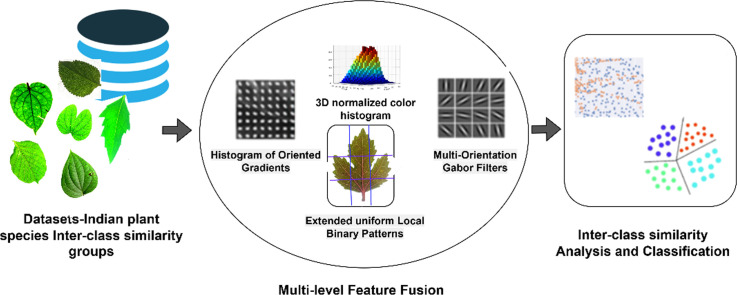

## Related research article

Pushpa, B. R., Rani, N. S., Chandrajith, M., Manohar, N., & Nair, S. S. K. (2024). On the importance of integrating convolution features for Indian medicinal plant species classification using hierarchical machine learning approach. *Ecological Informatics, 81*, 102,611.

Link: https://www.sciencedirect.com/science/article/pii/S1574954124001535?via%3Dihub

## Specifications table

**Table:** 

**Specification**	**Details**
Subject Area	Machine Learning, Computer Vision, and Botany
More Specific Subject Area	Indian Medicinal Plant Species Classification
Name of Method	CISCS: Classification of Inter-Class Similarity based Medicinal Plant Species Groups with Machine Learning
Name and Reference of Original Method	Gabor features, color features, LBP
Resource Availability	https://data.mendeley.com/datasets/748f8jkphb/3 B R, Pushpa; Rani, Shobha (2023), “Indian Medicinal Leaves Image Datasets”, Mendeley Data, V3, doi: 10.17632/748f8jkphb.3

## Background

Identifying plant species is essential for biodiversity conservation, ecological monitoring, and preserving traditional medicinal knowledge. Within this broader context, automatically recognizing Indian medicinal plants presents a particularly tough challenge. Many of these species look strikingly similar, while the same species can display subtle differences depending on environmental or geographical conditions. Added to this are practical hurdles such as limited labeled data, imbalanced datasets, and inconsistent imaging conditions—all of which make reliable classification even more difficult.

Earlier approaches have primarily relied on handcrafted features—like color histograms (RGB, HSV), texture descriptors (e.g., Local Binary Patterns), or simple shape-based measures (Hu and Zernike moments). While these methods can perform reasonably well when species are visually distinct, they often fall short when applied to medicinal plants, where the differences between species are extremely fine-grained. What is missing in much of the existing research is combining multiple complementary feature types—color, texture, and shape—into a single framework that can capture the full complexity of these plants.

Deep learning models, particularly convolutional neural networks (CNNs) such as ResNet18 and VGG16, have recently achieved remarkable success in image classification tasks by automatically learning rich visual features. However, their effectiveness depends heavily on large, balanced datasets and significant computational resources. In the case of medicinal plants, where data are scarce and species often share highly similar visual traits, CNNs are prone to overfitting and struggle to generalize well.

To overcome these challenges, our work introduces a multi-level fusion feature model that is both computationally efficient and effective for fine-grained classification. Combining the strengths of diverse feature types, the framework is designed to handle the subtle morphological differences and data limitations that define medicinal plant recognition.

In short, accurately classifying Indian medicinal plants remains an important yet underexplored challenge at the intersection of computer vision and machine learning. Our research seeks to bridge this gap by developing a domain-adapted classification framework that can reliably distinguish between closely related species by integrating advanced feature learning and robust classification strategies. The specific objectives of this work are as follows.

Design of a domain-adapted multi-granularity feature fusion that holistically integrates a diverse yet complementary suite of visual descriptors. Specifically, the use of normalized 3D color histograms, extended uniform local binary patterns (LBP{*P* = 24, *R* = 3} to encode fine-grained local textural information, and multi-orientation Gabor filters to capture discriminatory textural and Histogram of Oriented Gradients (HOG).

To rigorously evaluate the efficacy of the proposed framework on a meticulously curated dataset of Indian medicinal plant species exhibiting significant visual overlap—a critical real-world scenario that remains largely unaddressed in the current state-of-the-art. This targeted application will underscore the novelty and practical utility of the model in a demanding botanical identification task.

To apply a soft-voting ensemble classifier comprising Support Vector Machines (SVMs), Random Forests (RFs), and Gradient Boosting Machines (GBMs), augmented by Synthetic Minority Over-sampling Technique (SMOTE) to mitigate the adverse effects of class imbalance and ensure equitable model training. Furthermore, to introduce a cosine similarity-based inter-class relationship matrix to:

The proposed research problem formulation lays the groundwork for developing a sophisticated computational solution that advances the state-of-the-art in machine learning applications within plant sciences and offers a scalable and interpretable tool with broader implications for ecological research and biodiversity informatics within the Indian subcontinent.

Significant advancements were reported in the area of Artificial Intelligence (AI) and deep learning (DL) towards the classification and identification of medicinal plants [[1], [2], [3], [4], [5], [6], [7], [8], [9], [10], [11]]. Numerous studies have investigated the development of leaf-based classification using deep learning for shape analysis [1,2], shape and texture combinations [3], and machine learning methods [4]. Symbolic models [5] and deep convolutional neural networks (DCNNs) [6] were employed in recent works based on machine learning frameworks using leaf images [7], shape features with silent points [8], SVM and SMO with morphological features [9], CNNs for cotton leaf disease [10], and transfer learning for species classification [11].

Research by Tran Phat et al. [12] analyzed Vietnamese medicinal plant classification using zero-shot and incremental learning. Then, transfer learning to identify Ethiopian medicinal plants [13], with VGG19, handheld sensors, and random forest for Himalayan medicinal plant classification [14], and U-Net for semantic segmentation of Azadirachta indica canopy cover [15,16].

Furthermore, an ML-based electrochemical fingerprinting platform [17,18] for identifying closely related medicinal plants, MTJNet [20], and attention-based feature extraction [21,22] have been investigated as approaches to the classification of medicinal plants. Subsequent works used UAVRS and YOLO models [23,24] to classify medicinal plant species; the AI algorithms were examined in [25] along with DL models for palm disease classification [26,28,29] and a quantitative inversion framework (OQIF) for UAV hyperspectral images [27]. Table 1 highlights the significant studies and their importance in the context of the present research problem.

**Table 1:** Comparative analysis of significant works related to plant species classification.

Group	Studies	Methodologies	Domain Focus	Gaps / Limitations
1. Traditional Feature-Based (Shape/Texture)	[1,3,5,9,18]	Shape, Modified LBP, SVM	General & Medicinal Plants	Limited to hand-crafted features, no deep learning or fusion
2. Deep Learning for Disease Detection	[2,4,8,16]	CNN, Vision Transformers	Crop/Leaf Disease Detection	Focused on disease, not species identification
3. Deep Learning for General Species ID	[6,11,13]	CNN, SWP-LeafNET	General / Medicinal Plants	No hybrid feature strategy, lacks interpretability
4. Specialized for Medicinal Plants	[5,10,[12], [13], [14],[19], [20], [21]]	LBP, Deep Learning, Attention Models	Regional Medicinal Plants	Region-specific, lacks comprehensive feature fusion or inter-class analysis
5. Novel Approaches / Modalities	[7,15,17]	Zero-shot ML, Spectral, Semantic Segmentation, Electrochemical	Various	Focused on novel modalities or trees; not comparable in visual feature scope

The state-of-the-art works emphasize the need for scalable and adaptable approaches to address dataset complexities and environmental variations in medicinal plant classification. Future research should focus on developing robust methods for classifying medicinal plants with inter-class similarities and high accuracy, overcoming the limitations of current techniques. Thus, the present study aims to propose algorithmic interventions that can enrich artificial intelligence-based technology to identify different medicinal plant species. This work utilizes medicinal plant species that have been used for centuries, with the potential for treating many ailments and diseases. The automatic classification is carried out by using computer vision and machine learning techniques. A hierarchical classification scheme is used in this work to classify Indian medicinal plant species.

### Method details

**Fig. 1:**
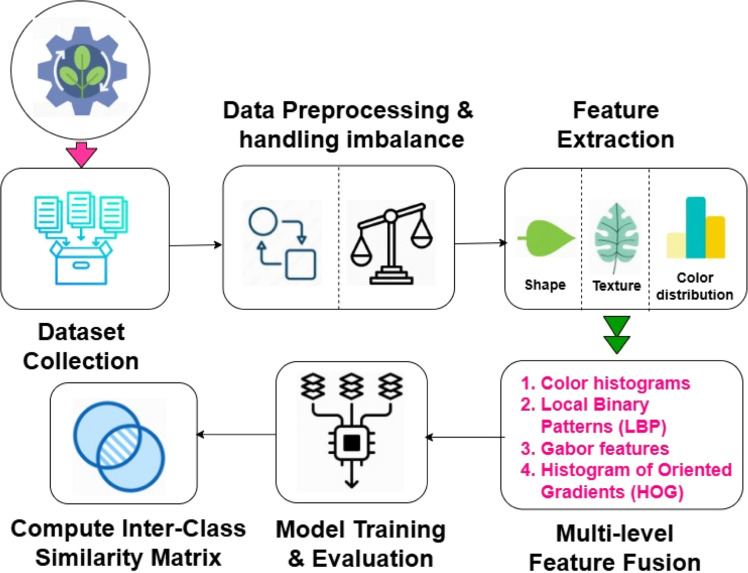
End-to-end workflow of the proposed method with aspects of multi-level feature fusion and improved data pre-processing pipeline.

The proposed approach employs an enhanced feature extraction procedure paired with a rigorous data pre-processing strategy to address the challenges of classifying plant species exhibiting high inter-class similarity. The key novelty of the proposed method lies in the seamless integration of multi-level feature fusion techniques, which effectively capture diverse visual feature patterns from color, texture, and shape descriptors, combined with the Synthetic Minority Over-sampling Technique (SMOTE) to address data imbalance. A comprehensive evaluation is conducted using a multi-level feature fusion technique to assess the performance of state-of-the-art classifiers, including SVM, Random Forest, K-Nearest Neighbours (KNN), Logistic Regression, Naïve Bayes, Decision Trees, Gradient Boosting, and AdaBoost. As a result, the proposed method aims to identify the most effective classification model by comparing its performance using multi-level feature fusion versus individual feature descriptors. Fig. 1 presents the workflow of the proposed method, highlighting the specific components.

### Dataset collection

The dataset comprises leaf samples of Indian medicinal plant species collected by Pushpa et al. [39] from various regions, including Mysore, Madanahalli, Bangalore, Mandya in Karnataka, and Kasaragodu in Kerala. The primary data collection was conducted at various botanical and medicinal gardens, nurseries, and farms, ensuring diverse environmental conditions and plant sources, including the Botanical Gardens of Chandravana Gardens, Govt. Ayurveda Medical College, Green Atmosphere Plant Nursery, Hebbal, Bhudevi Farms, Mysore, and Lalbagh Gardens, Bangalore. Additionally, samples are gathered from the Uppsala Medicinal Botanical Garden and the Medicinal Botanical Garden in Kasaragodu.

About sample collection and preparation, medicinal plant leaves were carefully collected and gently cleaned to eliminate dust and moisture. The sample preparation procedure ensures that the morphological characteristics of the leaves are preserved for feature analysis. Further, samples from plant species with similar morphological traits, such as leaf shape, venation patterns, and texture, are grouped and analyzed to study inter-class similarities. Five groups of plant species that reflect similar morphological characteristics are considered for experimentation. The details of these groups and the image acquisition method are discussed in a subsequent section. Table 2, Table 3 provide an overview of the dataset distribution and class details across various classes.

**Table 2:** Class-wise dataset distribution concerning various groups.

Group 1		Group 2		Group 3		Group 4		Group 5	
Class name	No. of samples per class	Class name	No. of samples per class	Class name	No. of samples per class	Class name	No. of samples per class	Class name	No. of samples per class
Balloon leaf	137	Brahmi	136	Amla	130	Betel	143	Aloevera	136
Bitter Guard	138	Dtura	137	Dill	134	Butterfly Pea	132	Bamboo	140
Castor	164	Hibiscus	132	Nagadalli	133	Giloy	136	Bermuda Grass	136
Coriander 1	148	Indian Borage	135	Nelanalli	149	Indian beech	135	Coriander 2	136
Gongura Papaya	138	Lantana	130	Shatavari	144	Insulin	139	Eucalyptus	138
Papaya	136	Neem	131	Tamarind	132	Malabar spinach	139	Ganagale	128
Prickly Poppy	138	Pumpkin	143	Tribulus Terrestris	145	Peepal	138	Ginger	131
		Rose	136	Wood Sorel	147	Pepper	137	Lemon grass	131
		Tridax Daisy	136			Taro	136	Mari Gold	132
								Onion	142
								Peel Kaner	145
								Tumbe	148

**Table 3:** Group-wise dataset distribution statistics.

Group	Number of classes	Average number of samples per class
Group 1	7	142
Group 2	9	135
Group 3	8	139
Group 4	9	137
Group 5	12	136

## Image acquisition setup

A customized image acquisition setup was employed, measuring 25 × 32 × 22 cm, prepared using cardboard and covered with white paper to ensure a uniform background. Then, an LED light source of 5 W was used to minimize external noise from ambient lighting and environmental disturbances. The top section of the setup was detachable and made of a white butter sheet for placing the leaves. The interior was designed to block external light sources effectively. The smartphone cameras with resolutions ranging from 13 MP to 64 MP, including models such as Xiaomi Mi 4i, Realme X2, Asus Zenfone Max Pro, and Realme GT Master, were utilized for image capture. This range of devices was chosen to avoid device-specific bias. Images were captured from each leaf's front and rear faces to capture divergent morphological and textural features. Images were taken at varying distances (10–20 cm) to simulate natural variations in imaging conditions. The images were acquired without camera filters or zoom options, and external light sources were turned off. Each Image was stored in .jpg format at a spatial resolution of 3120 × 4160 pixels.

To ensure dataset reliability, a comprehensive data cleaning pipeline was applied. Blurry, underexposed, overexposed, or occluded images were manually identified and removed. Duplicate or near-identical images were filtered using perceptual hashing techniques. Each Image was visually validated to confirm proper leaf placement and clarity. Preprocessing steps included resizing all images to a uniform input size of 224 × 224 pixels, normalization of pixel values, and background noise suppression through basic thresholding and cropping when necessary.

The dataset was also balanced for inter-device and intra-species variability by including leaves from multiple plants of the same species and at varying maturity stages. Each Image was annotated and grouped based on visual characteristics. Specifically, five species groups were defined: Group 1 consists of broad leaves with prominent venation; Group 2 includes narrow, delicate leaves; Group 3 features lobed leaf shapes; Group 4 encompasses smooth-edged leaves; and Group 5 represents complex venation with textured surfaces. Experts manually verified these groupings to ensure consistency.

**Fig. 2:**
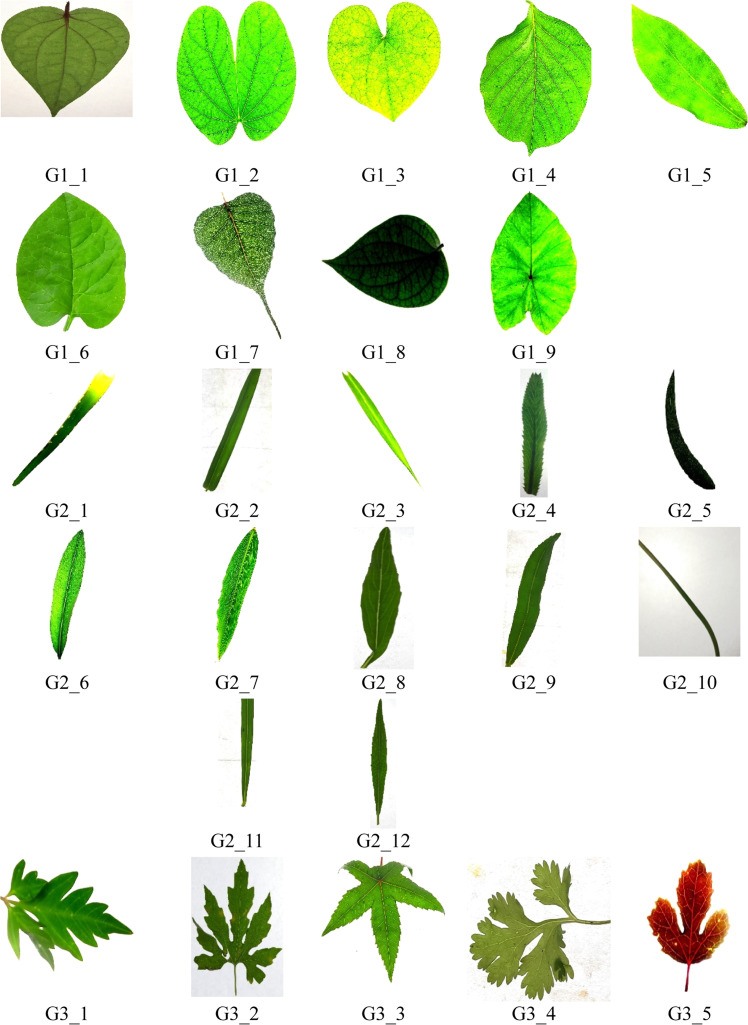

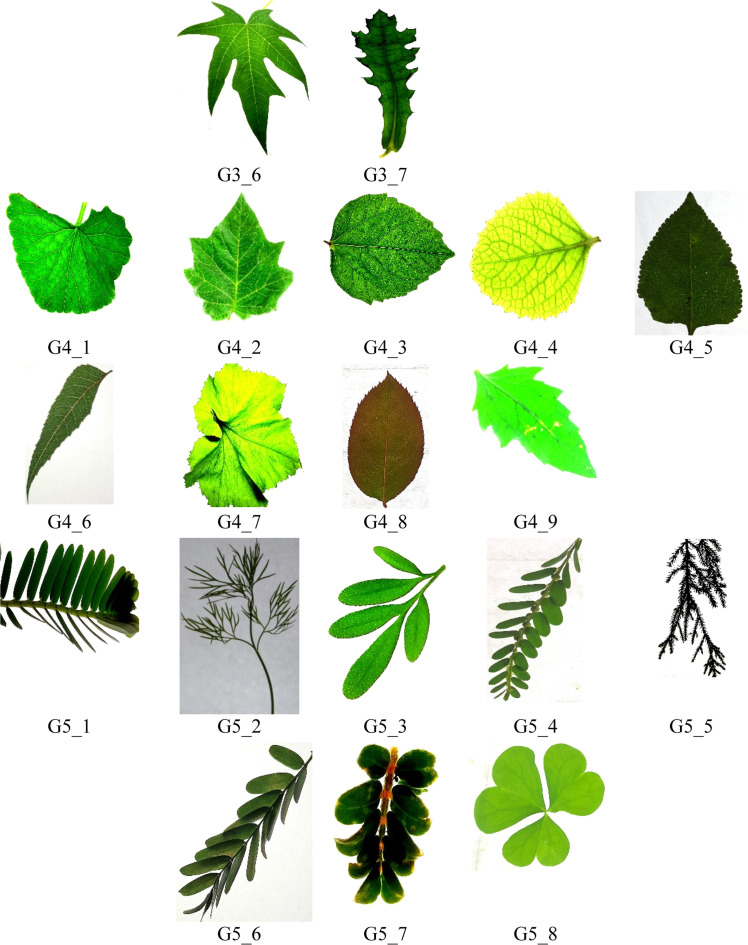
Representative samples from the dataset across Groups 1 to 5, showcasing inter-class similarities in leaf shapes, textures, and venation patterns. G1_1 to G1_9 belongs to group 1, G2_1 to G2_12 belongs to group 2, G3_1 to G3_7 belongs to group 3, G4_1 to G4_9 belongs to group 5, G5_1 to G5_8 belongs to group 5.

**Fig. 3:**
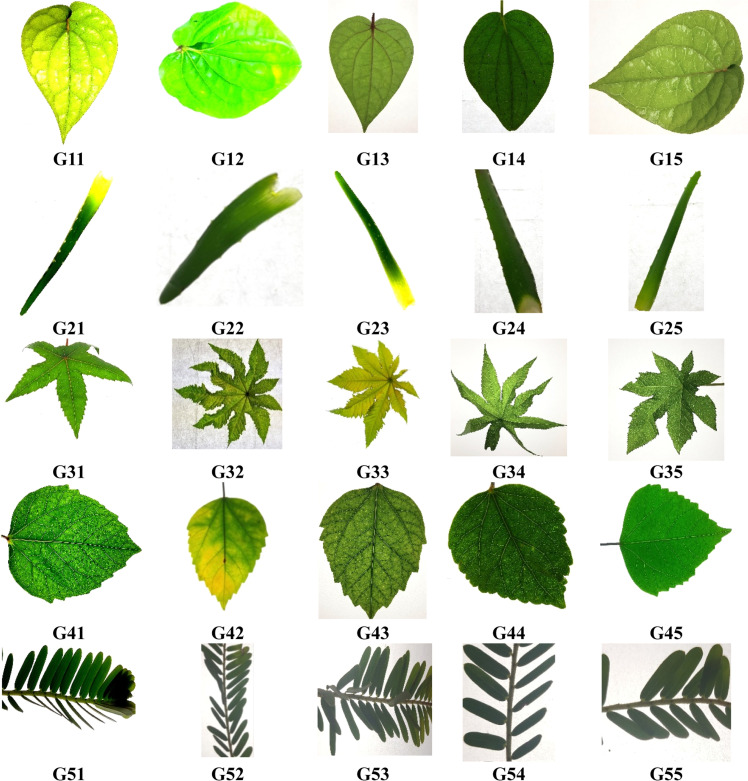
Representative samples from the dataset across groups 1 to 5, showcasing intra class variations in leaf shapes, textures, and venation patterns, G11 to G15 – group 1, G21 to G25 – group 2, G31 to G35 – group 3, G41 to G45 – group 4, G51 to G55 – group 5.

**Fig. 4:**
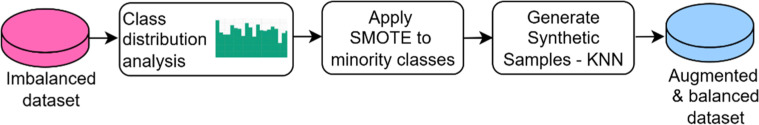
Data pipeline of handling imbalanced datasets using SMOTE.

For model training and evaluation, the dataset was randomly partitioned into training (70 %), validation (15 %), and testing (15 %) sets, ensuring class-wise balance across all subsets. Data augmentation techniques such as random rotations, horizontal/vertical flipping, brightness adjustments, and slight zoom variations were applied to enhance model generalization and mitigate overfitting. Fig. 2, Fig. 3 illustrate representative samples from the five defined groups, highlighting inter-class similarities and intra-class variations within the dataset.(Fig. 4).

### Pseudocode – proposed method

**Algorithm: MULTI-LEVEL-FUSION (D)**

**Table:** 

Input: Image dataset $D=\{I_{1},\;I_{2},\;\ldots I_{n}\}$
Output: Quantified performance metrics for classifiers
1 for each image I∈D do
2 Resize I→128×128; G←Grayscale(I)
3 $C_{1}\leftarrow 3D$ is a normalized RGB histogram
4 $C_{2}\;\leftarrow LBP\left(G;P=24,\;R=3,\;bins=26\right)$
5 $C_{3}\;\leftarrow \{\mu_{\theta };\;\sigma_{\theta }\;|\;\theta \;\in \left\{\;0^{0},\;45^{0},\;90^{0},\;135^{0}\right\}$ from Gabor(G)
6 $C_{4}\;\leftarrow HOG\left(G;cells=8\times 8,\;bins=9,\;norm=L2\right)$
7 $F\left(I\right)\;\leftarrow CONCAT\left(C_{1},\;C_{2},\;C_{3},\;C_{4}\right)$
8 end for
9
10 X←{F(I)|I∈D};Y←labels(D)
11 $\left(X^{\prime },Y^{\prime }\right)\;\leftarrow SMOTE\left(X,\;Y\right);\;X^{\prime }\leftarrow NORMALIZE\left(X^{\prime }\right)$
12 $Split\left(X^{\prime },Y^{\prime }\right)\;into\;\left(X_{train},\;Y_{train}\right),\;\left(X_{test},\;Y_{test}\right)$
13 Classifiers←{Adaboost,DT,GB,KNN,LR,NB,VM,RF}
14
15 for each M∈Clasisfiersdo
18 $M.fit(X_{train},\;Y_{train})$
19 $Y_{M}\leftarrow M.predict\left(X_{test}\right)$
20 Reportaccuracy(M),Precision(M),Recall(M),F1(M)
21 end for
22 Stop

The proposed methodology begins by resizing each image to 128×128 pixels and converting it to grayscale. Four feature descriptors are then extracted: a normalized 3D RGB color histogram, Local Binary Patterns (LBP) with *P* = 24, *R* = 3 and 26 bins, Gabor filter statistics (mean and standard deviation) at four orientations , and Histogram of Oriented Gradients (HOG) with $(0^{0},\;45^{0},\;90^{0},\;135^{0}$) 8 × 8 cells, 9 bins, and L2 normalization. These descriptors are concatenated to form the final feature vector. The resulting dataset is balanced using SMOTE and normalized before being split into training and testing sets. Eight classifiers AdaBoost, Decision Tree (DT), Gradient Boosting (GB), k-Nearest Neighbors (KNN), Logistic Regression (LR), Naïve Bayes (NB), Support Vector Machine (SVM), and Random Forest (RF)—are trained and evaluated, with performance measured using accuracy, precision, recall, and F1-score.

The implementation of the proposed feature extraction and classification framework, including the preprocessing steps, pseudocode, and evaluation scripts, is publicly available at: https://github.com/nshob1985/Image-classification-of-Indian-Medicinal-Plant-Species-with-Inter-class-similarities.git

### Data pre-processing and handling dataset imbalance

The image samples are subject to image resizing, label encoding, feature scaling, and, finally, addressing the issue of data imbalance using the SMOTE technique.

### Image resizing and label encoding

Let the original Image be represented by height h, width, and w, and the number of channels as c. In the proposed method, the Image is standardized to have dimensions 256 * 256 * 3, where c=3, h=256, and w=256. This resizing process ensures that every pixel P at location (i,j) satisfies the conditions 0≤i<256 and 0≤j<256. Thus, the image resizing transformation is represented as in (1).

(1) $$I_{h\times w\times c}\to I_{256\times 256\times 3}$$

The bilinear interpolation technique accomplishes the transformation in (1), where every pixel P at location (i,j) is transformed to determine its floating coordinates as =i*h/256, j=j*w/256. In bilinear interpolation, every pixel is computed as a weighted sum of four neighboring pixels, which includes leftat(I,j−1),rightat(I,j+1),topat(i−1,j),andbottomat(i+1,j)as given in (2).

(2) w(i,j)=left(i,j−1)+right(i,j+1)+top(i−1,j)+bottom(i+1,j)

Subsequently, the label encoding is performed as follows. Generally, machine learning models require numerical inputs. Therefore, it is important to convert categorical labels, such as plant species names, into unique integer labels. If $Labels=\{C_{1},C_{2}\;,\;C_{3}\ldots C_{k}\}$, then the mapping would result into Labels={0,1,2…k−1}.

### Handling dataset imbalance classes using SMOTE

In plant species classification, addressing dataset imbalance is critical, particularly when certain species possess low representation and remarkable visual similarities between types. SMOTE (Synthetic Minority Over-sampling Technique) is employed following the feature extraction process where every Image is converted into a high-dimensional feature vector. The method expands training data while enabling models to identify strong boundaries between species with overlapping visual patterns.

If $F^{d}$ is the feature space of a plan image p with das the dimensionality of the feature vector and the underrepresented plant species are denoted by N feature vectors, as in (3).

(3) $$U_{min}=\left\{x_{1},x_{2},\;x_{3}\ldots x_{N}\right\},\;\text{where}\;\text{each}\;x_{i}\epsilon F^{d}$$

For each sample $x_{i}$ of the underrepresented plant species set $U_{min}$, the s nearest neighbors are determined using the Euclidean distance technique as given in (4).

(4) $$NN\left(x_{i}\right)=\left\{x_{i}^{\left(1\right)},x_{i}^{\left(2\right)},\;x_{i}^{\left(3\right)}\ldots x_{i}^{\left(s\right)}\right\}$$

Subsequently, the synthetic samples are generated for the nearest neighbor $x_{i}^{\left(j\right)}\in NN\left(x_{i}\right)$ where 1≤j≤s using the linear interpolation technique in SMOTE. The linear interpolation process is repeated for every $x_{i}^{\left(j\right)}$ of the underrepresented class group samples to increase the number of samples relative to the majority sample classes. Thus, the SMOTE helps reduce the bias toward plant species classes and ensures the training process with abundant training images. Especially in the context of high inter-class similarity, many plant species share similar leaf shapes, textures, and color distributions and have underrepresented classes, which would lead to failure to capture the subtle distinctions among features of plant species. Thus, generating additional synthetic samples can effectively address the issue of underrepresented species in the feature space and help the classifier learn more discriminative feature boundaries.

### Feature extraction

The proposed method employs feature extraction techniques to capture features related to the leaf sample's color, texture, and shape, which is crucial to computing the subtle inter-class differences. The various techniques employed are discussed subsequently.

#### 3D Normalized color histogram with enhanced channel correlation

To effectively capture the variations inherent in morphologically similar plant species, we employ a 3D normalized color histogram. This technique transcends conventional channel-wise histogram computation by preserving the crucial inter-channel correlations within the Red, Green, and Blue (RGB) color space. The formulation involves discretizing the 3D color cube into a predefined number of bins and quantifying the joint probability distribution of pixel intensities across the three channels. Normalization ensures robustness to variations in image illumination and scale. This approach yields a holistic and discriminative color feature vector, effectively addressing the representational limitations associated with independent, marginal channel histograms.

If an image I of height h, width w, and number of channels c=3 is considered, then for each channel, a histogram comprising b bins is computed based on the range of intensity levels [0,255] as in (5) with a histogram function $Hist_{c}\left(b\right)$.

(5) $$Hist_{c}\left(b\right)=\sum_{\left(x,y\right)\in I}1\left\{I_{c}\left(x,\;y\right)\in Int_{s}\right\}\;and\;s=1\ldots b$$

$I_{c}\left(x,\;y\right)$ is the intensity at pixel (x,y) in channel c, and 1{.} is the indicator function. Further, the normalized histogram is computed as shown in (6).

(6) $$NC_{hist}=\frac{1}{h\times w}\left[h_{1}\left(1\right)\ldots h_{1}\left(b\right),h_{2}\left(1\right)\ldots h_{2}\left(b\right),h_{3}\left(1\right)\ldots h_{3}\left(b\right)\;\right]$$

In (6), $NC_{hist}$ is the normalized histogram vector.

The proposed enhancement ensures the preservation of relative pixel density across channels, which is crucial for identifying subtle differences in pigmentation among species and improved inter-channel interaction, allowing the model to capture color blending effects that are more relevant to real-world plant imagery. Thus, the novel application of 3D normalized histograms contributes significantly to the model’s ability to differentiate plant species with high visual similarity—particularly in the Indian medicinal plant domain, where color distinctions are often nuanced and critical to accurate identification.

#### Enhanced local binary patterns (LBP) with extended neighborhood

Local Binary Patterns (LBP) is a widely used texture descriptor that encodes the local neighborhood structure of each pixel into a compact binary representation. While standard LBP captures local texture effectively, its performance diminishes in complex visual domains, such as medicinal plant species classification, where finer texture details are crucial to distinguish morphologically similar classes.

To overcome this, we employ an enhanced LBP configuration that extends both the number of neighbors *P* and the radius *R*, enabling the capture of more contextual spatial information and multi-scale textures. Specifically, we adopt uniform LBP with *P* = 24 and *R* = 3, which significantly increases the texture descriptor’s sensitivity to subtle variations in leaf venation, surface roughness, and edge granularity.

LBP is one of the texture descriptor techniques that encode the local neighborhood of each pixel with comprehensive feature representation in Image I. The function of LBP applied on a pixel at location (x, y) is given by (7).

(7) $$LBP(I\left(x,\;y\right)=\sum_{nn=0}^{nn=P-1}S\left(I_{nn}\left(x,\;y\right)-I\left(x,\;y\right)\right).2^{nn}$$

In (7), nn represents the number of neighbors considered in the neighborhood, $I_{nn}\left(x,\;y\right)$ is the coordinates of the $nn^{th}$ neighbor, and S(t) is the thresholding function as elaborated in (8).

(8) $$S\left(t\right)=\left\{ \begin{array}{c} 1,\;if\;t\geq 0\\ 0,\;Otherwise \end{array}\right.$$

The feature space comprising LBP properties for the entire Image is obtained after the computation of the LBP of all the pixels, which defines the texture features as shown in (9).

(9) $$F_{LBP}=\left\{LBP\left(x_{1},y_{1}\right),\;LBP\left(x_{2},y_{2}\right)\ldots LBP\left(x_{r},y_{c}\right)\right\}\;\text{for}\;\text{an}\;\text{image}\;I\;\text{of}\;\text{dimensions}\;r\times c$$

Where $F_{LBP}$ represents the comprehensive LBP feature vector of the entire Image.

The enhanced neighborhood configuration (*P* = 24, *R* = 3) enables the extraction of richer and more granular texture patterns, especially in regions with delicate leaf structures. This improvement is particularly impactful in distinguishing between closely related plant species with subtle textural differences, a key novelty in Indian medicinal plant classification.

#### Gabor filter responses with multi-orientation texture representation

Gabor filters are powerful tools for texture analysis, as they simulate the response of human visual cortex cells to specific frequencies and orientations. In traditional applications, they are used with a limited number of orientations or fixed parameters, which may not be sufficient for fine-grained classification tasks involving visually similar classes.

To overcome these limitations, this work introduces a multi-orientation Gabor filter framework adapted to extract frequency-domain texture features from highly overlapping plant species images. The proposed approach captures directional textures and spatial frequency components that are otherwise lost in conventional texture models by applying Gabor filters across multiple orientations and scales. In the proposed method, multiple orientations (θ = 0°, 45°, 90°, 135°) and scales (σ values ranging from 1 to 3) were used to capture frequency textures robustly. Experiments with fewer orientations resulted in loss of detail in texture-rich samples, affecting classification accuracy negatively by ∼3 %.

Gabor filters are used extensively by many investigations to capture the texture features that are computed based on the Gaussian kernel. For pixels(x,y)of Image I, the response g is obtained by (10).

(10) $$g\left(x,\;y;\lambda ,\theta ,\psi ,\sigma ,\;\gamma \right)=exp\left(-\frac{x^{{}^{\prime }2}+\gamma^{2}y^{{}^{\prime }2}}{2\sigma^{2}}\right)cos\left(2\pi \frac{x^{\prime }}{\lambda }+\psi \right)$$

In (10), $x^{\prime }=xcos\theta +ysin\theta \;and\;y^{\prime }=-xsin\theta +ycos\theta$, λ is the wavelength, θ is filter orientation, ψ is the phase offset, σ is the standard deviation of Gaussian kernel function with γ is the spatial aspect ratio. Then, the response of the Image I using the Gabor filter is computed via convolution using filters of varying scales and orientations, as given by (11).

(11) $$R\left(x,\;y\right)=\left(I*g\right)\left(x,\;y\right)=\sum_{\left(i,j\right)}I\left(i,\;j\right)g\left(x-i,\;y-j\right)$$

Finally, the mean and standard deviation of the Gabor filter responses are computed to define the final feature vector $F_{Gabor}.$

(12) $$F_{Gabor}=\{mean(R\left(x,\;y\right)\cup standard\;deviation\left(R\left(x,\;y\right)\right\}$$

A multi-orientation filter bank is not used in prior studies that use fixed orientations; we employ a comprehensive set of Gabor filters with multiple orientations to encode texture directionality, which is vital for distinguishing leaf surfaces and venation in high-similarity species. The proposed Gabor feature extraction captures directional textures that remain consistent across varying lighting and image scales—common issues in real-world plant datasets.

Gabor filters in this work are uniquely tuned for Indian medicinal plant images, where species often exhibit subtle, frequency-based texture differences not well captured by spatial-domain features alone. Thus, enhanced Gabor response configuration, when integrated with color and shape features, significantly boosts the discriminatory power of the proposed fusion model, making it better suited for complex ecological classification tasks.

#### Histogram of oriented gradients (HoG) with fine-grained shape representation

Histogram of Oriented Gradients (HOG) is a prominent feature descriptor used to capture local shape and edge structure within images. While conventional HOG is often used in general object detection tasks, its full potential in fine-grained biological classification remains underutilized—especially in domains where structural similarity between classes is high, such as in Indian medicinal plant species.

To enhance shape-based differentiation in visually overlapping categories, our method adopts a fine-resolution HOG framework optimized explicitly for detecting subtle structural cues like leaf contours, serrations, and venation directions, which are often species-specific yet visually nuanced.

HoG features are crucial in understanding the shape of the leaf as it captures the gradient directions on a per-pixel basis in Image I. The Gradient of a pixel P at location (x,y) is computed as given by (13).

(13) $$Grad_{r}=I\left(r+1,\;c\right)-I\left(r-1,c\right)$$

(14) $$Grad_{c}=I\left(r,\;c+1\right)-I\left(r,c-1\right)$$

In (13) and (14), r and c indicate the row and column positions and $Grad_{x}$ and $Grad_{y}$ signify the gradient directions. Further, the gradient magnitude and orientation are given in (15).

(15) $$\text{The}\;\text{gradient}\;\text{magnitude},mag\left(r,c\right)=\sqrt{Grad_{r}{\left(r,\;c\right)}^{2}+Grad_{c}{\left(r,\;c\right)}^{2}}$$

(16) $$\text{The}\;\text{gradient}\;\text{orientation},\theta \left(r,\;c\right)=arctan\left(\frac{G_{y}\left(r,c\right)}{G_{x}\left(r,c\right)}\right)$$

In the proposed method, Imagethe I is divided into cells of size 8×8, which is subject to the computation of histograms of gradient orientations into b bins. Let $H_{cell}$ be the histogram of the cell in Image I, which is given by (17).

(17) $$H_{cell}=\sum_{\left(r,c\right)\in cell}w\left(\theta \left(r,c\right),\;b\right)m\left(r,c\right)$$

In (17), w(θ(r,c),b) is a function that determines the gradient orientation to bin b. The final feature vector of an image is then obtained by concatenating the histograms specific to each cell as given by (18).

(18) $$F_{HoG}=\left[H_{cell1},\;H_{cell2}\ldots H_{cellk}\right]$$

Thus, using high-resolution 8 × 8 cell divisions, our HOG configuration captures minute structural features of leaves, such as margin details and local curvature, essential for distinguishing species with high visual similarity. Further, enhanced orientation sensitivity is accomplished by the use of multiple orientation bins, which improves directional sensitivity, enabling the model to recognize subtle differences in leaf edge flow and directional texture patterns. Finally, unlike general object detection tasks, our HOG implementation is customized for botanical images, contributing to the first-of-its-kind structured shape analysis in classifying Indian medicinal plant species. Together with color and texture features, this refined HOG representation plays a critical role in constructing a multi-modal, highly discriminative feature space that boosts the performance of the proposed classification framework.

Table 4 presents the parameter setting employed during experimentations to create a fusion feature extraction model.

**Table 4:** Feature extraction parameters used to create the proposed fusion model.

Feature Type	Parameter	Value/Setting	Rationale / Novelty
Color Histogram	Channels & bins	[8, 8, 8]	Balanced detail across RGB channels; forms the global color signature.
Local Binary Pattern (LBP)	Neighbors (P), Radius (R)	*P* = 24, *R* = 3	Extended uniform LBP captures rich micro-textures; more discriminative.
	Histogram Bins	26 bins	Matches uniform LBP patterns, improves texture selectivity.
Gabor Filters	Orientations	4 (0°, 45°, 90°, 135°)	Multi-directional texture encoding in frequency domain.
	Frequency	0.6	Empirically chosen to detect mid-level frequency patterns.
Histogram of Oriented Gradients (HOG)	Orientations	9	Captures fine-grained edge and shape orientation, crucial for morphological cues.
	Pixels per Cell	8 × 8	Localized gradient descriptor.
	Cells per Block	2 × 2	Provides context-aware normalization.
	Block Normalization	L2-Hys	Stabilizes feature vectors against lighting/contrast variations.

### Multi-level feature fusion

As discussed earlier, a multi-level feature fusion technique is applied to individual feature extraction methods to unite them into a combined feature vector. This comprehensive feature representation combines the unique strengths of each descriptor to enhance classification accuracy. The typical approach to classifying plant species based on leaf images includes multiple feature extraction stages within a multi-level approach. At the initial level, color features are extracted via histogram calculation representing pixel intensity distribution among RGB channels. The computation of texture features follows this. Consequently, LBP patterns are extracted based on neighborhood relationships by establishing pixel encodings. Then, Gabor filters are employed to analyze texture patterns at different scales and orientations through their filter response outputs. Finally, the shape features are extracted based on the edge and gradient information extracted through the HOG technique to describe plant species' leaf shape and structure.

**Fig. 5:**
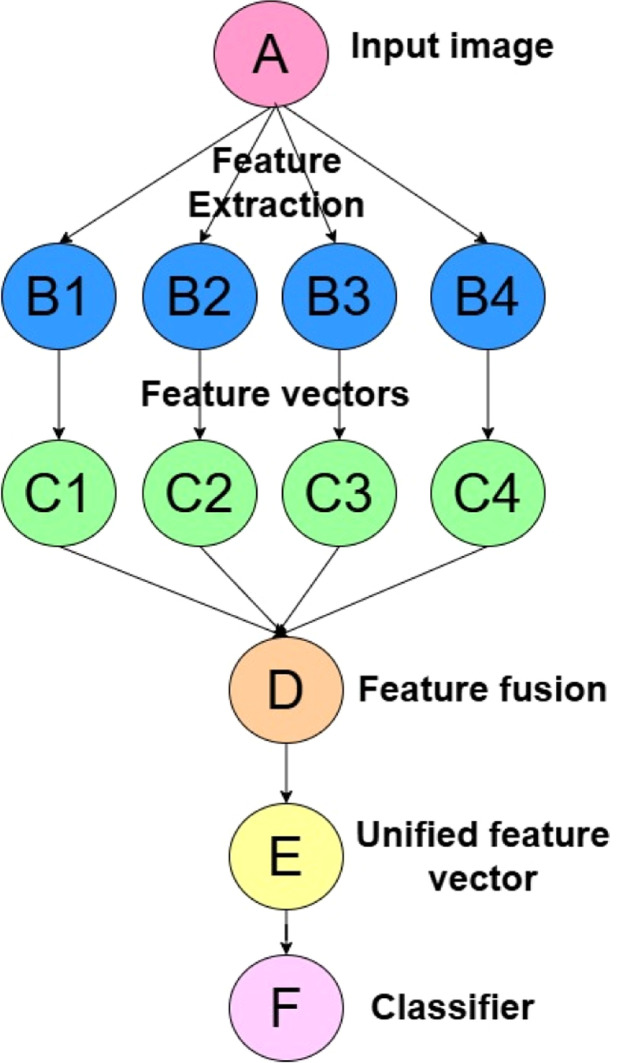
Multi-level feature fusion technique.

Thus, the multi-level feature extraction technique employs various extraction approaches to create multiple feature vectors combined into a unified extensive feature vector. The model benefits from a combined representation of information by considering the complementary feature patterns, leading to better species discrimination. Let input image (A), color histogram extraction (B1), LBP (B2), Gabor filter extraction (B3), HoG (B4), feature vectors obtained through four feature extraction methods be C1, C2, C3, and C4 producing unified, comprehensive feature vector resulting into feature fusion (D), to produce a final unified feature vector (E) that encapsulates all complementary feature patterns required to distinguish between plant species. This unified feature vector is then used for classifier training (F), as shown in Fig. 5.

Based on Fig. 5, in the proposed method for classifying plant species with inter-class similarities, the combined feature vector F is obtained by the fusion of feature vectors.

### Model evaluation

The proposed model training and evaluation aim to identify the robust classifier for plant species image classification specific to the context of groups of classes exhibiting inter-class similarities. The key novelty lies in devising an integrated approach that employs cross-validation techniques during the training and evaluation of the classifiers, utilizing the proposed unified feature vector.

The comprehensive feature vector F computed through a multi-level feature fusion process is fed into the classifier. We evaluated the model training using Adaboost, decision trees, Gradient boost, KNN, Naïve Bayes, logistic regression, Random Forest, and SVM classifiers in the proposed approach. Each classifier is finetuned to maintain interpretability and optimize time complexity for efficient classification.

### Ada boost (AB)

AB is a shallow tree-based sequential model that trains several weak classifiers. The model trains the samples by assigning larger weights to samples most likely to be misclassified by earlier models in the sequence. As a result, the final prediction is a weighted vote across a series of weaker machine-learning models. For each weak classifier $W_{c}\left(x\right)$ with an associated weight ω, the final updated weights of the classifier are given by (19).

(19) $$W\left(x\right)=sign\left(\sum_{c=1}^{T}\omega_{c}W_{c}\left(x\right)\right)$$

The final weights are updated iteratively based on the misclassification errors at each iteration. Generally, Adaboost focuses on identifying misclassified samples from the plant species with inter-class similarities.

### Decision trees (DT)

In the proposed method, leaf-based features related to the texture or shape patterns are extracted based on a particular curvature or color intensity range to interpret precise rules that align with linear distributions. Linear classifiers such as decision trees exploit the features computed recursively via binary decision rules using feature thresholds. Features and thresholds are chosen to classify the plant species at each node.

Given a feature vector, $F\in R^{d}$, decision rules are determined to form the tree as shown in empirical relation (20).

(20) $$if\;x_{j}\leq t\;then\;left\;branch\;else\;right\;branch\;$$

In (20), $x_{j}\;$is the $j^{th}$ feature, and t is the threshold. The decision tree classifier aims to maximize an impurity reduction metric such as Gini impurity or entropy, as in (21).

(21) $$\Delta I=I\left(p\right)-\left(\frac{n_{l}}{N}I\left(l\right)-\frac{n_{r}}{N}I\left(r\right)\right)$$

In (21), ΔI is the impurity reduction metric, p,l,andr are the parent, left and right nodes, $n_{l}$, $n_{r}$, and N represent the number of the nodes with class l and class r to the number of N.

### Random forest (RF)

The RF is an ensemble model that builds multiple decision trees by splitting the datasets into random subsets of data. The features are computed from data subsets through the aggregation of the predictions by the majority voting technique. Let V be the number of trees created producing the predictions $p_{1}\left(x\right),\;p_{2}\left(x\right),\ldots ,p_{V}\left(x\right),$ then the overall predictions P(x) is given by (22).

(22) $$P\left(x\right)=mode\left\{p_{1}\left(x\right),\;p_{2}\left(x\right),\ldots ,p_{V}\left(x\right)\right\}$$

The final predictions of random forests are achieved by averaging the predictions produced by multiple trees. This averaged prediction reduces variance, thereby improving the generalization towards predictions when dealing with leaf samples that exhibit noise.

### Gradient boost (GB)

GB is also an ensemble model that operates stage-wise, where each model is trained to reduce the errors from a combined previous prediction. In gradient boost, the initial model produces the prediction as $p_{0}\left(x\right)$ at stage 1, and then $p_{1}\left(x\right)$ is the combined prediction of stage 1 and stage 2. Similarly, the final stage produces a combined prediction obtained from all the stages by previous models. At stage m, the model $C_{m}\left(x\right)$ is updated as in (23).

(23) $$P_{m}\left(x\right)=P_{m-1}\left(x\right)+\nu .w_{m}\left(x\right)$$

Where $w_{m}\left(x\right)$ is the weak learner trained on the residual outcomes $re_{i}^{k}=P_{i-1}^{k}\left(x\right)-P_{i}^{k}\left(x\right)$ and ν is the learning rate, $P_{i-1}^{k}$ is the predictions obtained at iteration k by the(i−1) classifier model. The gradient boost model could model complex relationships that exist between the feature patterns associated with the problem of inter-class similarity classes,

### K-Nearest neighbour (KNN)

KNN is the best machine learning model to effectively capture the local structure in feature distributions, making it suitable for inter-class similarities among various classes. The KNN model is a non-parametric classifier that classifies a sample based on the majority voting of the K nearest neighbors. Consider a sample X; the Knearest neighbors can be determined through the technique of Euclidean distance as in (24). The predicted class $\hat{y}\;$is given by the mode of the K nearest neighbors as given by (25)

(24) $$dist\left(X-X_{i}\right)=∥X-X_{i}∥$$

(25) $$\hat{y}\left(X\right)=mode\left\{y_{\left(1\right)},y_{\left(2\right)},\ldots ,y_{\left(K\right)}\right\}$$

Where $y_{\left(1\right)},y_{\left(2\right)},\ldots ,y_{\left(K\right)}$ are the classes associated with K's nearest neighbors.

## Naïve Bayes (NB)

The classifier NB works based on the assumption of class conditional independence, which is usually violated by image-based features. Naïve Bayes produces the best performance with limited datasets and without any noise. The classifier works based on the Bayes theorem that computes the posterior probability of each class of the feature vector. The model chooses the class with the highest probability as given by (26) for a feature vector $F=\{f_{\left(1\right)},f_{\left(2\right)},\ldots ,f_{\left(d\right)}\},\;$and class C.

(26) $$P\left(C/F\right)\propto P\left(C\right)\prod_{i=1}^{d}P\left(f_{\left(i\right)}/C\right)$$

The final predicted class is given by (27).

(27) $$\hat{C}=\arg max_{c}P\left(C\right)\prod_{i=1}^{d}P\left(f_{\left(i\right)}/C\right)$$

## Logistic regression (LR)

LR is a linear model applied to handle binary or multiclass classification problems to estimate the probability of class membership function. For a feature vector F, the probability of the class is given by (28).

(28) $$P\left(y=1|F\right)=\sigma \left(W^{T}F+b\right)=\frac{1}{1+e^{-\left(W^{T}F+b\right)}}$$

Where W is the weight vector, and b is the bias. The proposed method evaluates the fusion feature vector to validate the existence of linearly separable properties. Logistic regression is a model that exhibits optimal performance by producing probabilistic outputs.

### Support vector machines (SVM)

In the presence of classes with high inter-class similarity issues, the SVM model captures the subtle differences between the features by mapping the features from higher to lower dimensional space. SVM employs the hyperplane in the feature space through a kernel trick; the Radial Basis Function (RBF) is employed in the proposed method. The decision function for SVM is given by (29).

(29) $$f\left(X\right)=sign\left(\sum_{i=1}^{N}\alpha_{i}y_{i}(K\left(X,X_{i}\right)+b\right)$$

Where $\alpha_{i}$ is the learned coefficient, $y_{i}$ is the class label, and K is the kernel function with b as the bias. Table 5 presents the parameter setting employed for classifiers throughout the experimentations.

**Table 5:** Parameter specifications of classification models used in the proposed study.

Model	Key Parameters	Value
SVM	Kernel, C, gamma	RBF, *C* = 10, gamma=0.1
RF	n_estimators	100
KNN	n_neighbors	5
LR	max_iter	1000
NB	–	–
DT	Criterion, max_depth, min_samples_split, random_state	gini, None, 2, 42
GB	n_estimators, learning_rate, max_depth, random_state	100, 0.1, 3, 42
AB	n_estimators, learning_rate, random_state	50, 1.0, 42

### Computation of inter-class similarity matrix

In the proposed classification task, similarity is computed between plant species in each group to understand the underlying challenges in classifying plant species with inter-class similarities. In this section, the procedure of computing inter-class similarities is described. Post classifier training, a similarity matrix is computed to identify the plant species that are causing ambiguities due to high visual similarity in their leaf features. The inter-class similarity matrix demonstrates how similar one class is to the other in the high-dimensional feature space produced by our multi-level feature extraction pipeline.

Initially, a mean feature vector is calculated for each class in the training datasets if D represents the training dataset divided into k classes where {1,2,3…k}∈j whose feature vectors are designated as given by (30).

(30) $$X_{j}=\left\{f_{1}^{\left(j\right)},f_{2}^{\left(j\right)}\ldots f_{nj}^{\left(j\right)}\right\}\;with\;f_{i}^{\left(j\right)}\in R^{d}$$

Where njrepresents the number of samples of a particular class j and d is the dimensionality of the feature fusion space. Subsequently, the mean feature vector $\mu_{j}\;$of each class j is computed as in (31).

(31) $$\mu_{j}=\frac{1}{n_{j}}\sum_{i=1}^{n_{j}}f_{i}^{\left(j\right)}$$

Then, in the next step, cosine similarity $C\_S_{pq}$ is calculated between mean vectors of two classes p and q as in (32).

(32) $$C\_S_{pq}=\frac{\mu_{p}.\mu_{q}}{∥\mu_{p}∥∥\mu_{q}∥}$$

Where $\mu_{p}.\mu_{q}$ is the dot product of mean vectors of classes p and q and $∥\mu_{p}∥$ is the Euclidean distance norm of $\mu_{p}$. The $C\_S_{pq}$ is quantized to [−1,1] and post-normalization to [0,1]. The similarity value close to 1 denotes that the classes are highly similar to each other, and values close to 0 indicate less similarity to each other. Finally, the inter-class similarity matrix $I_{s}$is computed as shown in (33).

(33) $$I_{s}=\left(\begin{array}{ccc} C\_S_{11} & \cdots & C\_S_{1k}\\ \vdots & \ddots & \vdots \\ C\_S_{k1} & \cdots & C\_S_{kk} \end{array}\right)$$

Thus, the similarity is calculated for all the pairs of classes ranging from{1,2…k}. The quantification of the inter-class similarity matrix $I_{s}\;$is helpful in the identification of pairs of classes with high similarity/dissimilarity to each other. The similarity matrix provides direct insights concerning the similarity of one class to the other. The high inter-class similarity implies that the current feature extraction may not capture the subtle differences between plant species. The observations from the inter-class similarity matrix would help refine the feature in the fused feature vector.

### Method validation

The methodology for the experiments was designed to evaluate the performance of classification models in identifying plant species from leaf images. The objective of experimentation is to address the issue of inter-class similarities and intra-class variations among five groups of medicinal plant species. The method validation process encompasses applying the fusion feature vector and baseline feature extraction methods individually to the machine learning classifiers to assess the efficiency of the proposed method. Method validation verifies that the proposed fusion feature vector is effective in classifying plant species, even towards unseen data. To perform the training, the dataset is divided into training, validation and test sets in the proportions of 70:15:15, and performance is evaluated using a K-fold cross-validation technique with ten folds. Then, the model is trained using K-1 folds and considering a different fold as the test set every time. Finally, the average of performance metrics is computed across K folds.

To test the model's generalization ability, the proposed fusion feature vector is evaluated across different subsets of the data. Further, the performance metrics such as accuracy, F1 score, precision, recall, and confusion matrix for each group of plant species are evaluated. Precision (34) emphasizes minimizing false positives and is valuable in scenarios where optimistic predictions are essential. Recall (35) prioritizes minimizing false negatives, ensuring that the maximum number of true positive cases are accurately identified. Similarly, accuracy (36) provides a clear and simple measure of the overall performance of the model, thereby determining the proportion of correctly predicted instances (both true positives and true negatives) out of the total instances in a dataset. Then, the F1-score (37) provides the optimal rate of prediction of the model, indicating the optimal balance between precision and recall. The precision, recall, and accuracy measures are quantified using TP (True Positives), FP(False Positives), and FN (False Negatives).TP of a given class are correctly identified samples by the model; FN are the samples misidentified by the model. FPs are the samples of a given class detected by the model but not considered as such by the expert. TN(True Negatives) are the medicinal plants not belonging to a given class as annotated by the expert and correctly identified as not belonging to a class by the model. A confusion matrix is a tabular representation that evaluates the performance of a classification model by illustrating the relationship between actual and predicted outcomes using TP, FP, FN,and TN.

### K-Fold cross-validation

5-Fold Cross-Validation is a widely used method for evaluating the robustness of a machine learning model by testing its performance on different subsets of the dataset. The cross-validation works on the following basis.

Step 1: The dataset D is divided into *k* = 5 equal folds (parts) $D_{1},\;D_{2}D_{3}D_{4},\;D_{5}$.
Step 2: For each Fold k
Step 3: $D_{k}$ is used as test data
Step 4: Train data $D_{T}=D-D_{k}$ (remaining 4-fold) is used train data
Step 5 Training on $D_{T}$
Step 6: Compute the performance metric $M_{k}$
Step 7: Compute the cross-validation performance
  $$CV_{p=\;}\frac{1}{k}\sum_{i=1}^{k}M_{i}$$

$CV_{p}$ is the cross-validation performance, *k* = 5 is the number of folds, and $M_{i}$ is the metric computed in the i^th^ fold. Metric $M_{i}$ can be P,R,AandF as in Eq. (34) to (37).

(34) $$P_{i}\;=\frac{TP_{i}}{FP_{i}}=\;\frac{CM_{i,i}}{\sum_{j}CM_{i,j}}$$

(35) $$R_{i}\;=\frac{TP_{i}}{FN_{i}}=\;\frac{CM_{i,i}}{\sum_{j}CM_{j,i}}$$

(36) $$A_{i}\;=\frac{TP_{i}}{FN_{i}+TP_{i}+FP_{i}}=\;\frac{CM_{i,i}}{\sum_{j}CM_{i,j}+\sum_{j}CM_{j,i}-CM_{i,i}}$$

(37) $$F_{1i}=\frac{2XP_{i}XR_{i}}{P_{i}+R_{i}}$$

I represent the actual class identified as rows of the matrix CM, and j represents the predicted class identified as columns of CM. The cell $CM_{i,j}$ indicates the number of instances from the actual class I predicted as class j.

### Method validation and analysis

The validation procedure is grouped into multiple phases in the proposed method. The objective of each phase is to establish the system's robustness via varying validation aspects to ensure reliable and accurate plant species classification. To ensure that the evaluation is unbiased and that the model is tested on unseen data, the dataset is partitioned into a training set and test set while preserving stratification with in-class distribution by adapting to K-Fold cross-validation.

The classifier's performance is quantitatively assessed using aggregate metrics such as accuracy, precision, recall, and F1-score. Additionally, the visualization of classifier-wise performance towards each group is depicted using performance curves such as ROC and precision-recall curves. These curves illustrate the trade-off between true positive and false favorable rates and also highlight the balance between precision and recall in situations of class imbalance. Finally, the performance of the proposed fusion feature method is compared with state-of-the-art methods to ensure robustness. Then, finally, the error analysis and post-hoc inter-class similarity among classes with groups are investigated along with confusion matrix analysis.

### Stress testing for group-specific robustness analysis

This section comprehensively explains the efficiency of various classifiers with the proposed fusion feature model to highlight the challenges towards group-specific plant species. Through ablation study, we intend to study the differences in classifier behavior in the context of plant species groups that exhibit inter-class similarities in leaf features.

In the proposed method, a novel performance quantification technique is adapted to understand and emphasize the efficiency of each classifier using an improved feature extraction model. The objective is to examine the robustness of classifiers with a dataset employed for the study comprising plant leaves that are captured in varying natural illumination conditions where the resilience of the model is tested in various real-world conditions. The stress testing of classifiers includes noise robustness, scale invariance, and rotation invariance.

In noise robustness, for an image I(x,y), we add noise component n(x,y) to obtain noisy Image $I_{n}\left(x,y\right)$ as in (38)

(38) $$I_{n}\left(x,y\right)=\;I\left(x,y\right)+nI\left(x,y\right)$$

In (38), n(x,y) is employed to simulate the noise caused by illumination variations. Through the ablation study on noise robustness, we intend to determine the optimal to determine the optimal noise threshold below which the proposed method shows robustness against noisy inputs. Subsequently, the testing is performed to interpret scale invariance, where varying sizes/scales of images influence the robustness of the proposed model. The different scales considered for evaluation include 64×64, 128×128, and 256×256. The scaling transformation 'S' is applied on the original image 'I' to obtain scaled Image I_s_ as in (39).

(39) $$I_{s}=S\left(I\right)*S_{f}$$

In (39), ‘S_f_' is the resizing /scaling factor; stress testing is also carried out to understand the rotation invariant performance of classifiers at angles at 90°, 180°, and 270°. For an Image 'I,' a rotation transformation 'R' is applied with the angle of '***θ***’ to contain rotated image ‘I_r_’ as given by (40)

(40) $$I_{R}=I_{\left(x,y\right)}*R\left(\theta \right)$$

**Fig. 6:**
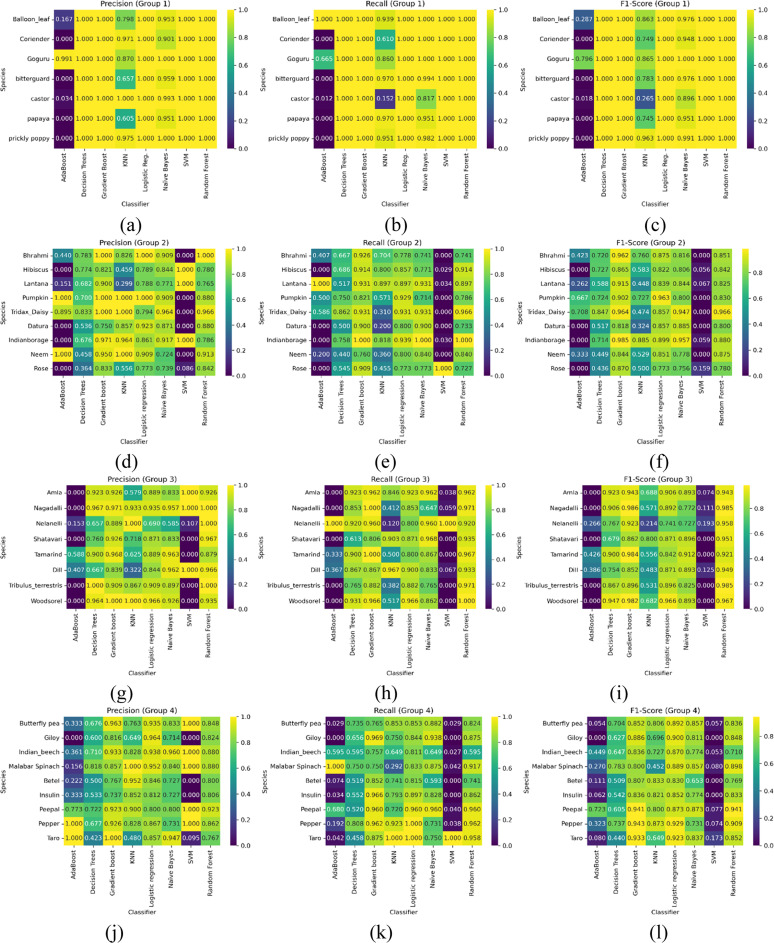

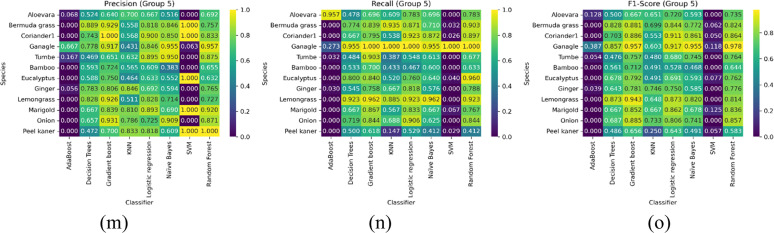
Classifier’s performance measures towards group 8 plant species (a) Precision – Group 1 (b) Recall – Group 1 (c) F1 Score – Group 1 (d) Precision – Group 2 (e) Recall – Group 2 (f) F1 Score – Group 2 (g) Precision – Group 3 (h) Recall – Group 3 (i) F1 Score – Group 3 (j) Precision – Group 4 (k) Recall – Group 4 (l) F1 Score – Group 4 (m) Precision – Group 5 (n) Recall – Group 5 (o) F1 Score – Group 5.

This procedure helps assess classifiers' performance concerning a given leaf orientation. The evaluation of the proposed enhanced fusion feature model towards Group 8 by various classifiers is presented in Fig. 6. The classifier’s performance is reported using standard performance metrics for each species within this group, such as precision, recall, and F1-score.

**Fig. 7:**
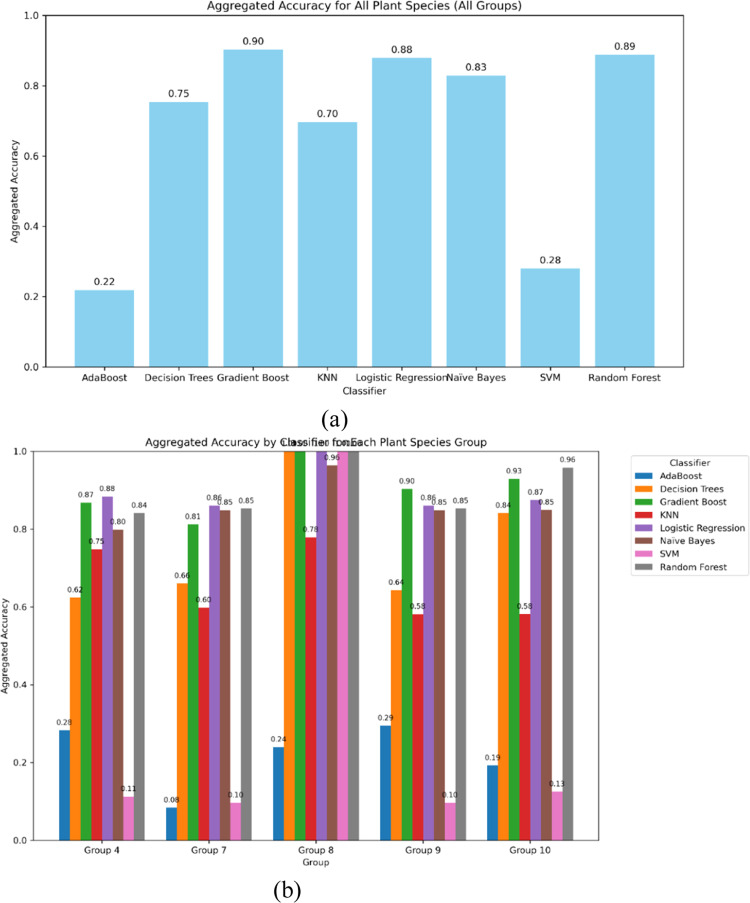
Aggregate accuracy of all plant species together with various classifiers.

Figs. 6 and 7, and Table 6 compare how each classifier performs on the classification of plant species with inter-class similarities in various groups. The ablation study in this section intends to evaluate the performance of different machine learning classifiers on five distinct groups of plant species. Each plant species group consists of medicinal, aromatic, and economically significant plants, classified using multiple algorithms to assess their robustness and generalizability. The classifiers considered for evaluation include AdaBoost, Decision Trees, Gradient Boosting, K-Nearest Neighbors (KNN), Logistic Regression, and Naïve Bayes. We quantify the performance by focusing on key metrics such as Precision, Recall, and F1-score to highlight the classifier's strengths and weaknesses.

**Table 6:** Aggregate performance metrics comparison using various classifiers – Group wise.

Group	AdaBoost ( %)	Decision Trees ( %)	Gradient Boost ( %)	KNN ( %)	Logistic Regression ( %)	Naive Bayes ( %)	SVM ( %)	Random Forest ( %)
Group 1	23.95	100.00	100.00	77.87	100.00	96.34	100.00	100.00
Group 2	29.46	64.34	90.31	58.14	86.05	84.88	9.69	85.27
Group 3	19.25	84.10	92.89	58.16	87.45	84.94	12.55	95.82
Group 4	28.29	62.40	86.82	74.81	88.37	79.84	11.24	84.11
Group 5	8.43	66.01	81.18	59.83	86.05	84.88	9.69	85.27

Group 1 includes commonly used medicinal plants such as Neem, Tulsi, Aloe Vera, and Ashwagandha, among others. These plants exhibit high morphological diversity, making classification challenging. Some key observations include that Gradient boost achieved the highest accuracy due to its strong feature selection and ensemble learning capability. The Naïve Bayes performed poorly due to its feature independence assumption, which does not hold for complex leaf textures and structures. Following this, KNN showed moderate results but struggled with species having similar leaf features.

Subsequently, the species in group 2, including aromatic and essential oil plants like lemongrass, eucalyptus, rosemary, and peppermint, were discussed. It was noticed that logistic regression performed exceptionally well, indicating that the plant features are linearly separable in this group. Then, Decision trees again struggled with plants with similar textures, such as lemongrass and citronella. Furthermore, AdaBoost achieved improved performance towards minority classes but failed to outperform Gradient Boosting.

In the study of group 3 species of leafy vegetable and nutritional plants, such as Coriander, Spinach, Fenugreek, and Amaranthus, that have overlapping morphological traits, the report obtained is as follows. It is noticed that KNN performed the best, suggesting that similarity-based classification works well for this group. Then, Naïve Bayes underperformed due to highly correlated features among species. Finally, Gradient boosting showed high recall, meaning it correctly identified many samples but had some misclassification errors.

Concerning group 4 of tree and woody plant species such as bamboo, teak, sandalwood, and mahogany, which share common structural properties, the key findings are as reported. It is inferred that Decision trees provided strong results, as the hierarchical nature of tree classification aligns well with the decision tree structure. Similarly, AdaBoost improved classification for rare species but struggled with feature similarity in particular species. Finally, Logistic Regression failed to generalize well due to nonlinear leaf and bark texture relationships.

Finally, the following observations were made in group 5 of herbs and shrubs, combining medicinal and wild shrubs like marigold, onion, Bermuda grass, and ginger. It is observed that Gradient Boosting emerged as the top performer due to its ability to handle diverse feature sets. Then, Naïve Bayes struggled due to high variance in leaf structure. Finally, KNN showed strong results for some species but failed where high inter-class similarity was present. In conclusion, the ablation study highlights that no single classifier performs best across all plant groups. Gradient Boosting and Decision Trees consistently performed well across different plant species, while Naïve Bayes and AdaBoost struggled in groups with highly overlapping morphological features. The findings suggest that ensemble-based and instance-based learning techniques are more suitable for complex plant classification tasks.

From the perspective of inter-class variations, the ablation study revealed that the importance of each feature set varied by species group. Groups with high inter-class similarity benefited more from the combined use of all feature types. In contrast, groups with more distinct visual differences were less sensitive to removing one feature set. Furthermore, tree-based classifiers (Decision Trees, Random Forest, Gradient Boost) and Logistic Regression maintained robust performance even with reduced feature sets. In contrast, classifiers like AdaBoost and SVM were more adversely affected by removing any feature set. KNN and Naïve Bayes exhibited moderate sensitivity, suggesting that their reliance on distance measures and probabilistic assumptions may be more impacted by the loss of discriminative information.

### AUC-ROC and PR curve analysis

**Fig. 8:**
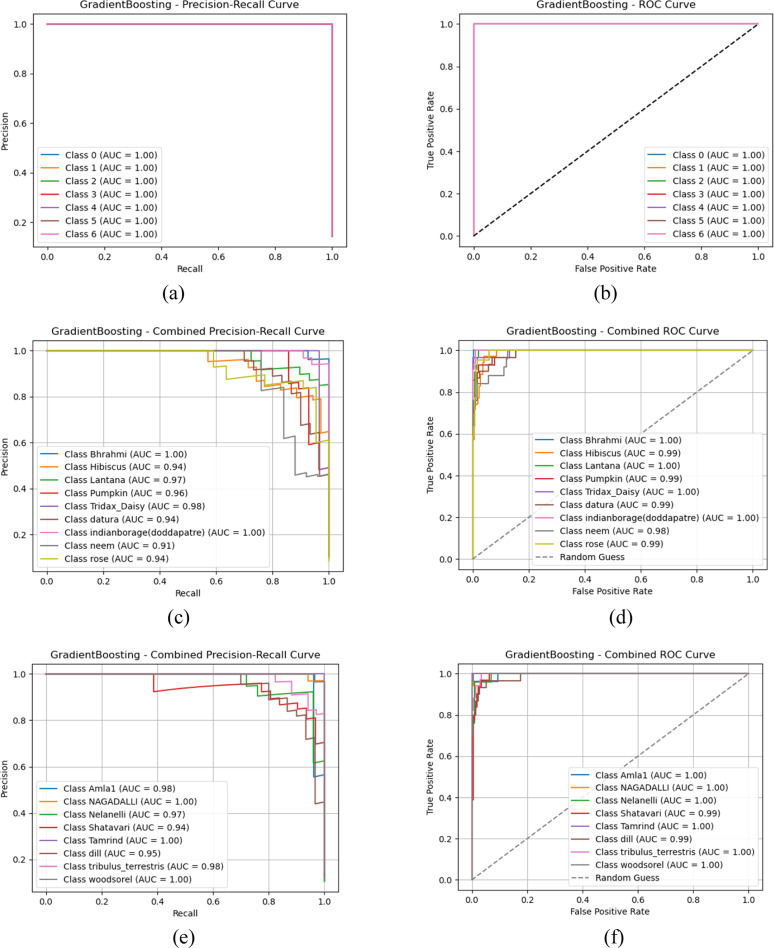

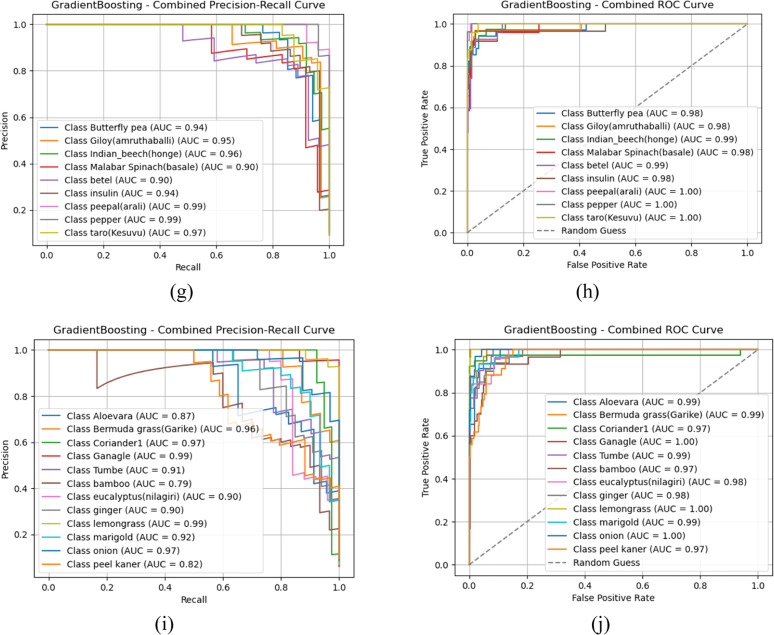
AUC-ROC and Precision-Recall curve analysis outcomes of Gradient boost classifier (a) Gradient boost-PR Curve- Group 1 (b) Gradient boost- AUC-ROC Curve- Group 1 (c) Gradient boost-PR Curve- Group 2 (d) Gradient boost- AUC-ROC Curve- Group 2 (e) Gradient boost-PR Curve- Group 3 (f) Gradient boost- AUC-ROC Curve- Group 3 (g) Gradient boost-PR Curve- Group 4 (h) Gradient boost- AUC-ROC Curve- Group 4 (i) Gradient boost-PR Curve- Group 5 (j) Gradient boost- AUC-ROC Curve- Group 5.

**Fig. 9:**
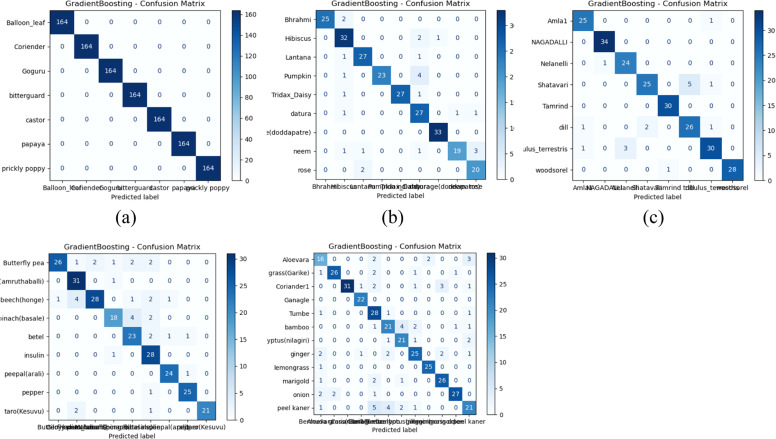
Confusion matrix representation of classifiers (a) Gradient boost –Group 1 (a) Gradient boost –Group 2 (c) Gradient boost –Group 3 (d) Gradient boost –Group 4 (e) Gradient boost –Group 5.

To thoroughly assess the classification performance of machine learning models with multi-level fusion feature models, we present an in-depth evaluation using Receiver Operating Characteristic (ROC) curves, Area Under the Curve (AUC) scores, and Precision-Recall (PR) curves. These metrics provide insights on how well each classifier distinguishes between plant species with inter-class similarities. The comparative performance of Random Forest and Gradient Boosting are presented, as these models demonstrated strong results across different groups. Specifically, AUC-ROC Analysis emphasizes the actual positive rate (sensitivity) against the false positive rate, with AUC indicating the classifier's ability to distinguish between classes. A higher AUC value suggests superior discrimination capabilities. Similarly, the Precision-Recall (PR) curve is especially useful in imbalanced datasets, showing the trade-off between precision (correct optimistic predictions) and recall (ability to capture all positive instances). Fig. 8 presents the detailed visualizations and discussions of the results for each group.(Fig. 9).

**Fig. 10:**
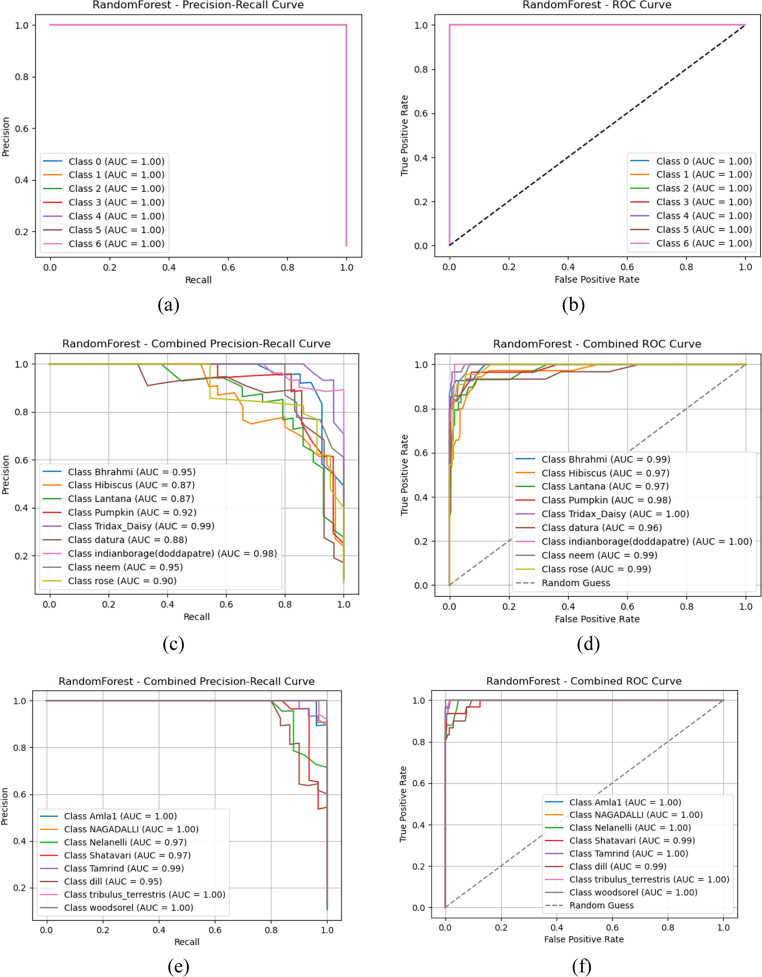

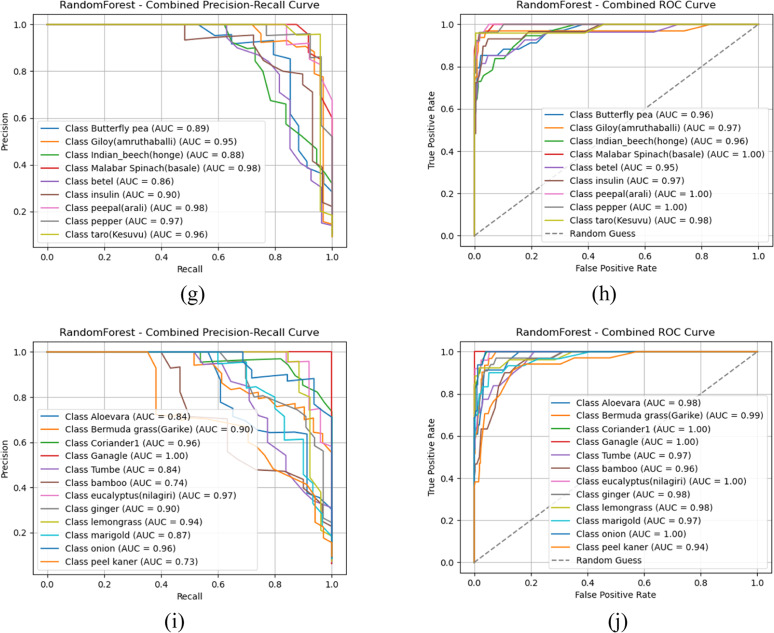
AUC-ROC and Precision-Recall curve analysis outcomes of Random Forest classifier (a) Gradient boost-PR Curve- Group 1 (b) Gradient boost- AUC-ROC Curve- Group 1 (c) Gradient boost-PR Curve- Group 2 (d) Gradient boost- AUC-ROC Curve- Group 2 (e) Gradient boost-PR Curve- Group 3 (f) Gradient boost- AUC-ROC Curve- Group 3 (g) Gradient boost-PR Curve- Group 4 (h) Gradient boost- AUC-ROC Curve- Group 4 (i) Gradient boost-PR Curve- Group 5 (j) Gradient boost- AUC-ROC Curve- Group 5.

**Fig. 11:**
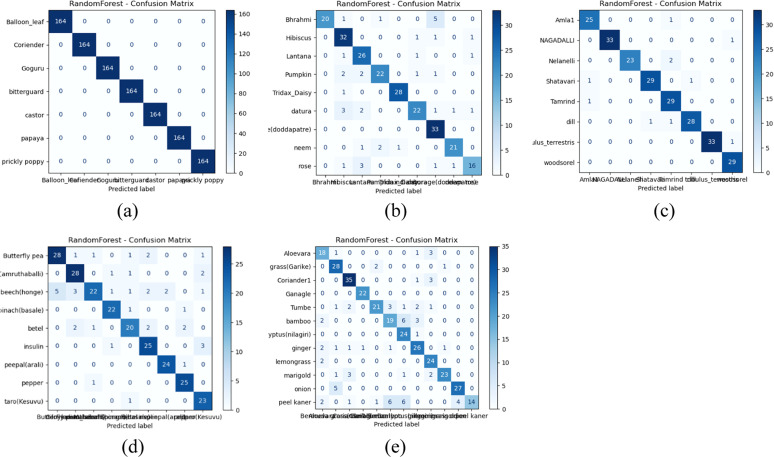
Confusion matrix representation of classifiers (a) Random Forest –Group 1 (b) Random Forest –Group 2 (c) Random Forest –Group 3 (d) Random Forest –Group 4 (e) Random Forest –Group 5.

Based on the observations from Fig. 8, Fig. 9, Fig. 10, both Gradient boost and Random forest achieve perfect classification towards classes in group 1 with AUC-ROC and Precision-Recall of 1. This indicates that both models can precisely distinguish all classes when the images are clean, well-structured, and contain no distortions. AUC-ROC and Precision-Recall values remain above 0.95 for each class in Gradient Boost, with at least one or more classes still maintaining nearly 1.0 performance. Then, Random Forest exhibits a slight drop in performance, about 0.20 to 0.25 lower than Gradient Boost. Thus, it helps us understand that Gradient Boost is more robust to moderate noise and distortions in the dataset, allowing it to retain high classification accuracy. Also, Random Forest is slightly more sensitive to noise, which leads to a minor performance reduction, likely due to feature inconsistency across decision trees in the ensemble.(Fig. 11).

Towards Groups 3 and 4, for Gradient Boost, it is nearly 1 for all classes, indicating that it effectively learns from the given features despite potential variations in data. This suggests that Gradient Boost generalizes well across different conditions and maintains strong discriminative power. Furthermore, Random Forest projects a slight reduction in AUC-ROC and PR scores for each class compared to Gradient Boost but still maintains strong overall classification ability. Hence, Random Forest is slightly more sensitive to dataset variations than Gradient Boost. It is inferred that both classifiers demonstrate robust learning capabilities for Groups 3 and 4, with Gradient Boost showing slightly stronger resilience to dataset variations; with respect to Group 5, Gradient Boost projects that three classes show AUC-ROC and Precision-Recall values in the range of 0.84 to 0.87, while the remaining classes retain values above 0.95 or close to 1.0. This indicates that some classes are more challenging to classify in this group, possibly due to overlapping features, intra-class variations, or noise interference. From the perspective of Random Forest performance, there is a similar trend but with a further decrease compared to Gradient Boost for each result. This suggests that Random Forest struggles to distinguish certain classes in more complex scenarios. Both classifiers show a performance drop, but Gradient Boost remains more stable in handling class imbalances or feature similarities.

## Computation of inter-class similarity matrix

**Fig. 12:**
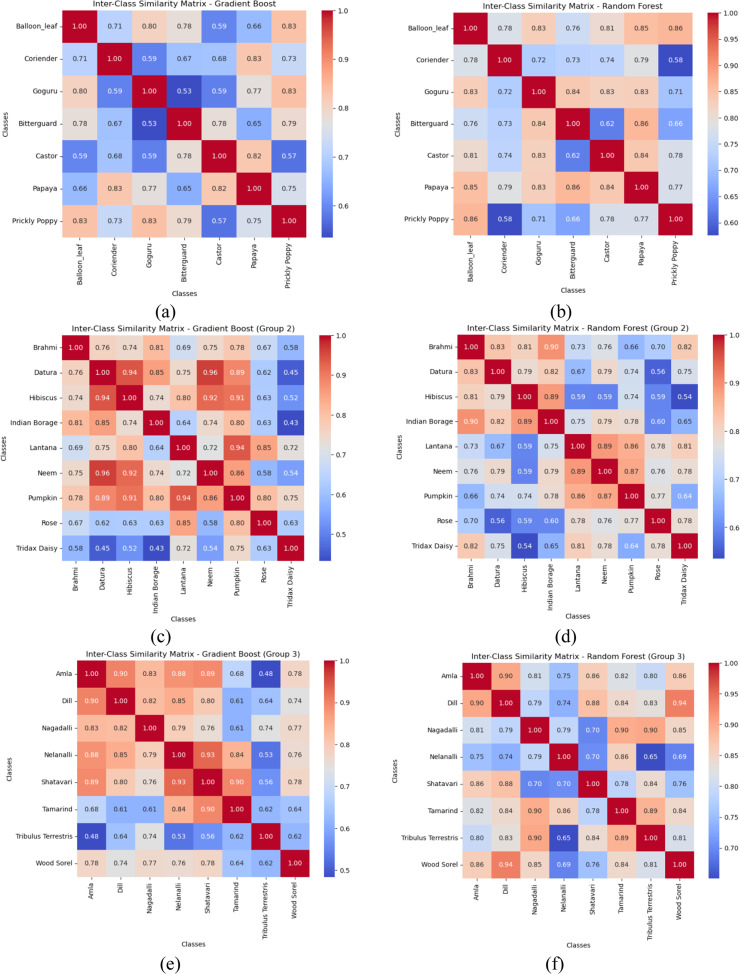

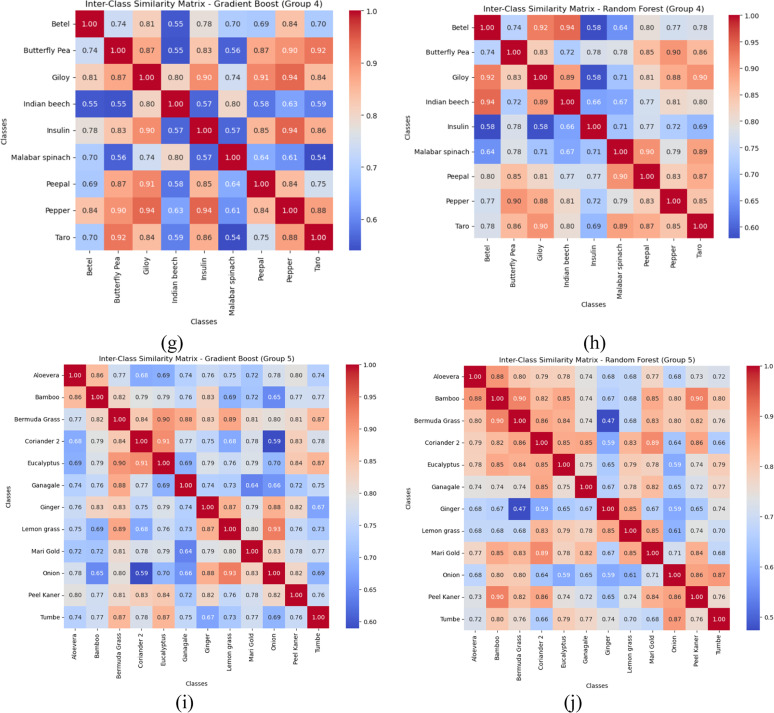
Inter-class similarity matrix of best-performing classifiers (a) Gradient boost-Group 1 (b) Random forest – Group 1 (c) Gradient boost-Group 2 (d) Random forest – Group 2 (e) Gradient boost-Group 3 (f) Random forest – Group 3 (g) Gradient boost-Group 4 (h) Random forest – Group 4 (i) Gradient boost-Group 5 (j) Random forest – Group 5.

The proposed study explores the inter-class similarities between plant species for each of the five groups by employing distance-based analysis using the fusion feature model similarity. Adapting to the feature importance derived from high-performing classifiers such as Gradient Boost and Random Forest classifiers, we quantify the relationships from one class to the other using cosine similarity. The obtained similarity matrices are visualized as heatmaps to clearly depict class relationships. The values close to 1 signify close feature alignment and, thus, the potential for misclassification. By analyzing these heatmaps, we aim to pinpoint closely related species that may benefit from the proposed enhanced fusion feature model. This leads to the evaluation of the efficacy of our feature extraction methods and an understanding of how high inter-class similarity impacts model performance. Finally, the similarity matrices help us better organize the feature space, enabling us to finetune the fusion feature model to achieve improved classification accuracy. Fig. 12 depicts the inter-class similarity matrices for the Gradient boost and Random Forests classifiers.

The inter-class similarity matrices computed based on the Gradient Boost and Random Forest classifiers reveal critical feature relationships about the classification performance. Notably, the high cosine similarity values imply classes with indistinguishable feature representations. This specific aspect is often observed in medicinal plants sharing similar morphological characteristics. Conversely, well-separated class clusters are also evidenced by low inter-class similarity scores, which help the model achieve better classification rates. The Random Forest exhibits slightly higher similarity values than Gradient Boost, suggesting that Gradient Boost has a superior ability to capture subtle feature variations. The high similarity among multiple classes across classifiers results in the necessity for developing a fusion features model based on shape descriptors, texture, and color descriptors. Thus, these findings helped us propose informed model refinement strategies, including dataset augmentation through synthetic data generation, to improve classification accuracy. Regarding medicinal plant classification with high inter-class similarities, these insights emphasize the need for techniques to mitigate the risks inherent in misclassifications. Thus, the similarity matrices offer a quantitative measure to guide the development of more accurate and robust classification systems for plant species with inter-class similarities.

## Stress testing

In the proposed study, machine learning models are evaluated under standard conditions with clean and well-structured data provided in the dataset. However, real-world deployment poses challenges, such as noise, image transformations, and distortions, which can significantly impact model performance. Therefore, to assess the resilience of the classifiers, we conduct a stress testing analysis, where models are tested under different challenging conditions, as mentioned in the previous section. In this section, Table 7 presents accuracy results under these conditions, offering insights into how each classifier adapts to different stress factors. This analysis aims to identify classifiers that maintain stability across multiple conditions and determine their robustness ranking based on performance degradation.

**Table 7:** Accuracy with Noise, Scaling, and Rotation.

Condition	AdaBoost ( %)	Decision Trees ( %)	Gradient Boost ( %)	KNN ( %)	Logistic Regression ( %)	Naïve Bayes ( %)	SVM ( %)	Random Forest ( %)
No Stress (Clean Images)	23.95	100.00	100.00	77.87	100.00	96.34	100.00	100.00
Gaussian Noise	22.00	95.00	98.00	72.00	97.00	88.00	85.00	96.00
Scale 64×64	18.50	85.00	90.00	65.00	80.00	75.00	70.00	91.00
Scale 256×256	24.00	98.00	99.00	79.00	99.00	94.00	98.00	99.50
Rotation (90°)	21.00	90.00	92.00	70.00	85.00	82.00	78.00	94.00
Rotation (180°)	19.00	82.00	88.00	68.00	81.00	77.00	72.00	90.00

Furthermore, Table 8 provides classifier-specific observations, highlighting the best and worst-case performance scenarios while assessing stability across transformations. Thus, the study analyzes these results obtained under real-world conditions such as noise, size, and rotation. Through this, we can identify which classifiers are most suitable for real-world applications where data variability is inevitable.

**Table 8:** Classifier-Specific observations.

Classifier	Best Case for clean samples	Worst Case for occluded/rotated samples	Stability Across Conditions	Robustness Ranking
Random Forest	100 %	85 % (occlusion)	Consistent with minor drops	1st (Most Robust)
Gradient Boost	100 %	88 % (180° Rotation)	Handles noise, scaling well	2nd
Decision Trees	100 %	82 % (180° Rotation)	Moderate degradation	3rd
Logistic Regression	100 %	80 % (64×64 Scaling)	Affected by resolution loss	4th
KNN	77.87 %	65 % (64×64 Scaling)	Sensitive to transformations	5th
Naïve Bayes	96.34 %	75 % (64×64 Scaling)	High variance in performance	6th
SVM	100 %	70 % (64×64 Scaling)	Highly sensitive to noise, occlusion	7th (Least Robust)

The transformations applied to the dataset revealed distinct implications for classifier performance. Introducing Gaussian noise resulted in a general accuracy decline, with simpler models like Naïve Bayes and SVM experiencing the most significant degradation. Conversely, tree-based models, such as Random Forest and Gradient Boost, exhibited robustness, likely due to feature redundancy and ensembling techniques. Scaling the images down to 64×64 led to a substantial accuracy drop across all classifiers, particularly affecting Naïve Bayes and SVM, highlighting their dependence on high-resolution features. However, scaling up to 256×256 yielded minimal performance loss and even slight improvements in some classifiers, indicating the benefit of preserved feature detail. Finally, rotating images by 90° and 180° caused moderate accuracy reductions. SVM and Naïve Bayes were the most affected, emphasizing the importance of rotation-invariant features for models sensitive to spatial relationships.

From Table 8, Random Forest and Gradient Boost consistently demonstrated the highest robustness across the applied image transformations, effectively overcoming the impacts of noise, scaling, and rotation. Conversely, Naïve Bayes and SVM exhibited significant sensitivity, experiencing substantial accuracy declines under the same transformations. Scaling images down to 64×64 proved particularly detrimental, with SVM, Naïve Bayes, and KNN suffering the most significant performance losses. Occlusion (20 %) posed the most significant challenge among all transformations, resulting in the largest overall accuracy drop. Furthermore, rotation-based distortions adversely affected classifiers that rely on spatial dependencies, such as KNN and SVM, highlighting the importance of feature invariance for these models.

### Comparison of fusion feature model with individual feature extraction techniques

It is known that feature extraction plays a dominant role in determining the performance of classification models. Traditional methods such as color histograms, Local Binary Patterns (LBP), Gabor Filter Responses, and Histograms of Oriented Gradients (HoG) have been widely used in the literature. In the present work, we aim to leverage their complementary strengths to enhance feature representation vis multi-level fusion feature model. This section presents the comparative study of the fusion feature model with individual feature extraction techniques. Multiple classifiers are considered for evaluation, and performance metrics are assessed to determine the effectiveness of fusion-based feature representation. The results indicate whether the fusion approach offers significant advantages over standalone feature extraction methods. Table 9 presents the outcomes of the comparative analysis.

**Table 9:** Performance of traditional feature extraction techniques against a Fusion Feature Model.

Feature Extraction Method	Accuracy ( %)	Robustness to Noise	Computational Cost	Feature Discriminability	Suitability for Plant Species Classification
Color Histograms	78.5	Moderate	Low	Low (sensitive to illumination changes)	Useful for color-rich species but lacks texture information
Local Binary Patterns (LBP)	82.3	High	Moderate	Moderate (good for texture but limited spatial info)	Effective for capturing leaf texture but lacks shape details
Gabor Filter Responses	85.1	High	High	High (captures multi-scale texture details)	Works well for texture-based differentiation but computationally expensive
Histogram of Oriented Gradients (HoG)	80.7	Moderate	Moderate	High (captures shape and edge details)	Useful for leaf shape and edge-based classification
Fusion Feature Model (Combination of Above)	92.8	Very High	Higher but Optimized	Very High (leverages strengths of all)	Best performance due to multi-feature representation

From Table 9, it is noticed that comparing the multi-level fusion features with traditional feature extraction methods proves that Random Forest and Gradient boost are the best classifiers, even with individual feature extraction techniques. It is inferred that the combined feature patterns offers a comprehensive feature representation to classify the plant species with inter-class similarities. The individual strengths of each feature extraction technique are also included in Table 8. The performance comparison of traditional feature extraction techniques with Gradient boost classifiers toward various groups is depicted in Table 10.

**Table 10:** Classifier performance comparison for different feature extraction techniques.

Group	Color Histograms ( %)	LBP ( %)	Gabor Filters ( %)	HoG ( %)	Fusion Feature Model ( %)
Group 1	78.5	85.2	90.1	82.4	100.00
Group 2	72.3	79.8	84.6	76.5	85.27
Group 3	74.9	81.5	87.2	78.9	95.82
Group 4	69.5	76.2	82.9	74.1	84.11
Group 5	65.7	72.4	79.3	70.8	85.27

The analysis outcomes in Table 10 reveal that fusion features consistently yielded superior performance compared to individual techniques across all groups. Among individual methods, Gabor Filters and Local Binary Patterns (LBP) generally outperformed Color Histograms and Histograms of Oriented Gradients (HoG). Notably, Group 1 experienced the most significant performance gains from feature fusion, likely due to high inter-class similarities, where the combined information from multiple feature sets enhanced classification robustness. Conversely, Groups 4 and 5 exhibited the most minor improvements with fusion features, indicating that the species within these groups possess inherent features.

### Benchmarking with deep learning and traditional classifiers

To further validate the effectiveness of proposed multi-fusion feature model, we have conducted a comparative analysis with benchmark deep learning architectures VGG-16, MobilenetV2 and ResNet-18. The deep learning models were fine-tuned via transfer learning to classify group wise Indian medicinal plant species. The deep learning models were chosen by motivation of prior successes in plant classification tasks in researches reported such as Ayur-PlantNet by Pushpa and Rani [30], a lightweight CNN tailored for Ayurvedic species. Mulugeta et al. [31] using ResNet and VGG models. Additionally, Pushpa et al. [32] demonstrated the benefits of combining handcrafted and deep features in a hierarchical setup, which informed our fusion strategy. These studies collectively suggest that deep models provide strong baselines, but hybrid approaches can achieve comparable accuracy with reduced computational demands, making them suitable for low-resource hardware.

In this study, we employed ResNet18 and VGG16 to perform group-wise plant species classification. ResNet18 incorporates residual learning through identity skip connections, VGG16, combines 13 convolutional layers and 3 fully connected layers. These models were selected to represent a range of trade-offs between depth, parameter size, and computational complexity as presented in Table 11.

**Table 11:** Detailed specifications of deep learning architectures.

Model	# Layers	Key Modules	Layer Types	Total Params	Complexity (MACs)
ResNet18	18	Residual Blocks	Conv, BN, ReLU, Identity Skip Connections	∼11.7M	∼1.8 GFLOPs
VGG16	16	Sequential Conv + FC layers	Conv, ReLU, MaxPool, FC	∼138M	∼15.5 GFLOPs

We compared our deep learning results against standard machine learning classifiers including AdaBoost, Decision Trees, Gradient Boosting, K-Nearest Neighbours (KNN), Logistic Regression, Naive Bayes, SVM, and Random Forest (See Table 6). Accuracy ( %) and loss ( %) for each group is reported in Table 12, Table 13. Then, to train the deep learning models, hyper parameter specifications adapted includes 20 epochs using the Adam optimizer with a learning rate of 0.001, batch size of 32 with cross-entropy loss function. The input images were resized to 224×224 pixels to match with the input requirements of the deep learning architecture with data augmentations such as random horizontal flips and normalization during pre-processing.

**Table 12:** Group wise classification accuracy ( %) of deep learning models**.**

Group	ResNet18		VGG16	
	Train Accuracy ( %)	Validation Accuracy ( %)	Train Accuracy ( %)	Validation Accuracy ( %)
Group 1	97.34	94.29	97.65	96.73
Group 2	91.88	85.63	92.57	90.83
Group 3	98.00	95.50	93.87	92.00
Group 4	99.79	99.59	93.85	70.78
Group 5	79.01	73.99	95.96	77.33

**Table 13:** Group wise classification loss ( %) of deep learning models**.**

Group	ResNet18		VGG16	
	Train Loss ( %)	Validation Loss ( %)	Train Loss ( %)	Validation Loss ( %)
Group 1	8.64	20.69	20.50	26.48
Group 2	23.82	42.99	26.58	39.33
Group 3	08.25	19.74	52.24	43.22
Group 4	00.08	01.06	23.80	20.14
Group 5	34.18	38.36	50.05	43.81

According to Table 12, Table 13, the group-wise analysis emphasizes distinct performance trends between ResNet18 and VGG16. With regard to group 1, both models perform well, though ResNet18 exhibits lower training and validation loss, while VGG16 achieves slightly higher validation accuracy. For group 2, VGG16 outperforms ResNet18 in both validation accuracy and loss, indicating superior generalization in this category. Group 3 clearly favors ResNet18, which dominates in accuracy and loss, whereas VGG16 struggles with a notably high training loss, suggesting poor learning. The most dramatic difference appears in group 4, where ResNet18 nearly achieves perfect validation accuracy and minimal loss, while VGG16 experiences a significant drop in validation accuracy pointing to severe overfitting or underfitting. In group 5, both models face difficulty, but ResNet18 maintains more stable loss values. VGG16 again shows signs of overfitting, achieving high training accuracy but failing to generalize effectively in validation.

Furthermore, in comparison with proposed model outcomes as per Table 6, when comparing the ResNet18 and VGG16 with the proposed multi-level feature fusion, some interesting group-specific trends emerge. In group 1, traditional ML models like Decision Trees, Gradient Boost, Logistic Regression, SVM, and Random Forest all reached perfect accuracy (100 %), even outperforming the already strong deep learning results—ResNet18 at 94.29 % and VGG16 at 96.73 %. This clearly shows that ML models are particularly well-suited to this group. Moving to Group 2, the results are more closely matched. VGG16 and Gradient Boost both performed around 90 %, suggesting a slight edge for ML, although SVM lagged far behind with poor performance.

In group 3, ResNet18 and Random Forest were nearly neck-and-neck, both scoring around 95 %, while other ML models also performed well. This group appears balanced, with no clear winner. However, in group 5, ML models bounce back. Random Forest, Logistic Regression, and Gradient Boost all outperformed the deep learning models, suggesting they generalize better. Looking across all groups, ResNet18 consistently delivers strong, stable results, especially in more challenging data scenarios. On the other hand, the proposed approach particularly Random Forest, Gradient Boost, and Logistic Regression shows excellent generalization and holds its own against deep learning, especially when the data is structured or less complex. While VGG16 struggles with overfitting in some groups, most ML models maintain a solid balance between training and validation performance.

## Conclusion and future work

In this study, a novel multi-level fusion feature model is implemented to classify Indian medicinal plant species with high morphological similarities. By integrating advanced feature descriptors such as color histograms, Local Binary Patterns (LBP), Gabor filters, and Histogram of Oriented Gradients (HOG), we were able to capture the diverse characteristics of plant images. The proposed model demonstrated significant improvements in classification accuracy, towards plant species exhibiting high inter-class similarity. Additionally, an ensemble learning approach combined with SMOTE-based data augmentation is also designed to handle the issue of class imbalance. The ecological informatics on plant species is interpreted through cosine similarity-based relationship matrix.

The experimental results underscore the potential of proposed approach in effectively captures features with subtle distinctions between classes. Thus the proposed work contributes to the field towards important applications of biodiversity conservation, medicinal plant identification, and ecological studies. While the proposed model has shown promising results, several avenues exist for future improvement including refinement of the feature extraction process using deep learning to achieve even more discriminative features. Further, to explore the generalization of proposed model to other plant species datasets incorporating varying image quality. Use of transfer learning to leverage pre-trained models for improved performance with smaller datasets. Finally, deployment of proposed model in real-time edge computing applications.

## Limitations

While the proposed approach demonstrates strong performance in classification tasks, few limitations and dependencies exist to provide the best performance.

• Regarding computational complexity, the fusion feature model may need to further decrease the computational cost compared to individual methods.
• Training and inference require higher memory and processing power on resource-constrained devices.
• The varying illumination conditions, occlusions, image acquisition modality may impact the effectiveness of the proposed model.
• It is noticed that certain feature extraction techniques might degrade more significantly under stress conditions, affecting overall performance.
• The controlled image acquisition setup limits scalability and may not generalize well to real-world, unconstrained environments with natural backgrounds.
• Manual annotation and handcrafted group definitions introduce subjectivity and may not scale efficiently to larger, more diverse plant datasets.

## Ethics statements

Human subjects do not appear in our research work nor animal experiments or even social media data for that matter.

## CRediT authorship contribution statement

**N. Shobha Rani:** Conceptualization, Methodology, Data curation, Project administration, Formal analysis, Writing – original draft. **Bhavya K R:** Conceptualization, Methodology, Data curation, Supervision, Formal analysis. **I. Jeena Jacob:** Conceptualization, Methodology, Data curation, Supervision, Formal analysis, Writing – original draft. **Pushpa B. R:** Methodology, Data curation, Software, Formal analysis, Validation. **Bipin Nair BJ:** Methodology, Software, Formal analysis, Validation. **Akshatha Prabhu:** Methodology, Software, Formal analysis, Validation.

## Declaration of competing interest

The authors declare that they have no known competing financial interests or personal relationships that could have appeared to influence the work reported in this paper.

## Data Availability

Provided link to datasets used. Dataset is available at Mendeley in the work related to Indian Medicinal plant datasets is a repository that consists of medicinal plants images.
